# Examining the Impact of Simultaneous Alcohol and Cannabis Use on Alcohol Consumption and Consequences: Protocol for an Observational Ambulatory Assessment Study in Young Adults

**DOI:** 10.2196/58685

**Published:** 2024-09-25

**Authors:** Rachel L Gunn, Jane Metrik, Nancy P Barnett, Kristina M Jackson, Sharon Lipperman-Kreda, Robert Miranda Jr, Timothy J Trull, Mary Ellen Fernandez

**Affiliations:** 1 Center for Alcohol and Addiction Studies Department of Behavioral and Social Sciences Brown University Providence, RI United States; 2 Providence Veterans Affairs Medical Center Providence, RI United States; 3 Rutgers Addiction Research Center Rutgers Robert Wood Johnson Medical School Rutgers University Piscataway, NJ United States; 4 Pacific Institute for Research and Evaluation Berkeley, CA United States; 5 Psychological Sciences University of Missouri Columbia, MO United States

**Keywords:** ecological momentary assessment, alcohol, cannabis, consequences, transdermal alcohol biosensors, ambulatory assessment, mobile phone

## Abstract

**Background:**

There is significant conflicting evidence as to how using cannabis while drinking alcohol (ie, simultaneous alcohol and cannabis use) impacts alcohol volume consumed, patterns of drinking, and alcohol-related consequences. The impact of simultaneous use on drinking outcomes may be influenced by several within-person (eg, contextual) and between-person (individual) factors.

**Objective:**

This study was designed to examine naturalistic patterns of alcohol and cannabis use to understand how simultaneous use may impact drinking outcomes. The primary aims were to understand the following: (1) if simultaneous use is associated with increased alcohol consumption and riskier patterns of drinking, (2) if simultaneous use leads to increased alcohol consequences, and (3) how contextual circumstances moderate the impact of simultaneous use on consumption and consequences.

**Methods:**

Data collection involves a 28-day ambulatory assessment protocol in which a sample of non–treatment-seeking young adults who report simultaneous use of alcohol and cannabis complete ecological momentary assessments (random, event-contingent, and time-contingent surveys) of alcohol and cannabis use, contexts, motives, and consequences on their personal smartphones while continuously wearing an alcohol biosensor bracelet. Participants also complete a baseline assessment, brief internet-based check-in on day 14, and a final session on day 28. Community-based recruitment strategies (eg, social media and flyers) were used to enroll 95 participants to obtain a target sample of 80, accounting for attrition.

**Results:**

Recruitment and data collection began in May 2021 and continued through June 2024. Initial results for primary aims are expected in October 2024. As of March 2024, the project had recruited 118 eligible participants, of whom 94 (79.7%) completed the study, exceeding initial projections for the study time frame. Remaining recruitment will provide the capacity to probe cross-level interactions that were not initially statistically powered. Strengths of the project include rigorous data collection, good retention and compliance rates, faster-than-expected enrollment procedures, use of a novel alcohol biosensor, and successful adaptation of recruitment and data collection procedures during the COVID-19 pandemic.

**Conclusions:**

This is the first investigation to assess the key momentary predictors and outcomes of simultaneous use as well as self-reported and objective (via alcohol biosensor) measures of alcohol consumption and patterns. The results of this study will inform prevention efforts and studies of individuals who use cannabis who are engaged in alcohol treatment.

**International Registered Report Identifier (IRRID):**

DERR1-10.2196/58685

## Introduction

### Overview

Alcohol and cannabis use are among the most commonly used substances in the United States. Rates of using both substances are particularly high among young adults [1], who also report the highest rates of simultaneous alcohol and cannabis use (ie, using alcohol and cannabis at the same time so that the effects overlap) [2]. Young adults (ie, aged 18-26 years) are the only age group for whom rates of cannabis use increased following the legalization of cannabis [3]. Co-use of alcohol and cannabis (ie, use of both substances but not necessarily so that the effects overlap), and particularly simultaneous use (ie, use of both alcohol and cannabis so that the effects overlap [4]), are associated with increased risky behaviors and negative consequences, including driving under the influence, alcohol-related injuries, and other legal, academic, interpersonal, physical, and mental health problems relative to alcohol or cannabis use only [1,5-9]. Despite the increased risks associated with co-use, findings as to whether or not cannabis use leads to momentary increases in alcohol consumption or consequences remain mixed [10], and our understanding of how co-use confers susceptibility to alcohol problems remains poorly understood. It is critical to understand the impact of cannabis use on alcohol use and its consequences, as alcohol use is the third-leading cause of preventable death in the United States [11,12], and alcohol misuse cost US $249 billion in 2010 [13] and contributed to 5.1% of the global burden of disease and injury [14]. Globally, alcohol misuse was the leading risk factor for death and disability among those aged between 15 and 49 years in 2016 [15]. The present work was designed to use ambulatory assessment (AA) methods to closely examine the momentary impact of simultaneous use on alcohol consumption, patterns, and consequences, as well as the social and contextual factors that may moderate those effects.

### Simultaneous Alcohol and Cannabis Use

Laboratory work examining the acute effects of simultaneous use suggests that cannabis enhances the subjective effects of alcohol [16-18], increases motivation to drink alcohol [19,20], and leads to synergistic cognitive impairment [21-23] relative to when alcohol is used alone. Although these studies provide ideally controlled conditions for examining the acute effects of combined cannabis and alcohol, they are less ideally suited to examine complex contextual factors (eg, location, activity, and social context) and naturalistic patterns of consumption. Observational studies of simultaneous use in which participants self-report their patterns of alcohol and cannabis use yield a naturalistic observation of cannabis’ impact on alcohol outcomes. In particular, the use of repeated measurement, including the Timeline Followback (TLFB; calendar-assisted method using anchoring dates to gather substance use estimates at the day level) [24], and AA methods, including ecological momentary assessment (EMA), are increasingly used to measure fine-grained patterns and correlates of co-use at the event level [25]. Initial research suggests simultaneous use is associated with higher levels of alcohol and cannabis consumption relative to when either substance is used alone [26-29]. However, competing findings exist in an observational study of primarily college students who provided 14 days of daily diary entries suggesting no increased risk of consumption [30]. With regards to alcohol-related consequences, there is a body of literature suggesting a higher risk of consequences on simultaneous or co-use days relative to alcohol-only days (refer to the review by Lee et al [31]). Despite these significant findings, several studies found no significant association between simultaneous use and consequences, especially when alcohol or cannabis quantity is controlled [27,32-34].

These conflicting results about the effects of simultaneous use have given way to 2 competing theories, namely whether cannabis acts as a substitute (ie, replacing the effects of alcohol, resulting in decreased use) or a complement (ie, used to enhance the effects of alcohol, resulting in increased use) [10,35,36]. The extant empirical literature provides compelling evidence for both substitution and complementary effects, in addition to nuanced findings based on population and mechanisms, including patterns (eg, order of use in a day) and contexts (eg, social settings) of use. These seemingly conflicting findings highlight that simultaneous use is at times complementary and at other times reflects substitution patterns (ie, within-person effects). There is a need for additional research examining fine-grained details of simultaneous use events, including mechanisms and contexts of simultaneous use, to isolate the conditions in which simultaneous use may lead to increased consumption and consequences.

### Proposed Mechanisms of the Impact of Simultaneous Use on Alcohol Outcomes

#### Overview

**Figure 1:**
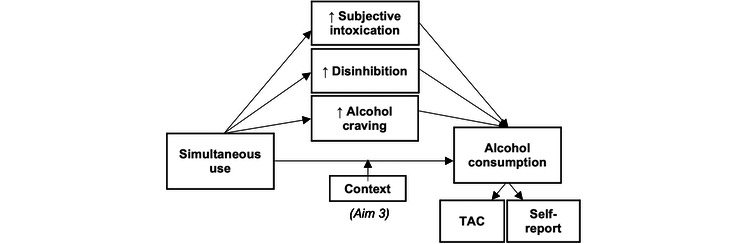
Aim 1: proposed mechanisms of the association between simultaneous use and alcohol consumption. TAC: transdermal alcohol concentration.

**Figure 2:**
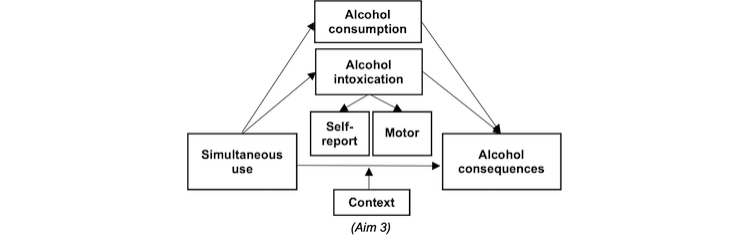
Aim 2: proposed mechanisms of the association between simultaneous use and alcohol consequences.

Incentive-sensitization theory posits that addiction results from the foundational characteristics of *drug liking* and *drug wanting* [37]. Expansion of this theory suggests that incentive sensitization, paired with loss of inhibitory control, (ie, disinhibition) leads to increased motivation to use substances [38,39]. We can test whether this theory applies to how simultaneous use events confer risk for increased alcohol consumption by examining whether simultaneous use indeed leads to increased motivation or craving, subjective effects, and reduced disinhibition, and, in turn, increased alcohol consumption and consequences. Each of these proposed mechanisms can be assessed with AA methods and are discussed in more detail subsequently (refer to Figures 1 and 2 and the Aim 1 section).

#### Intoxication

There is mixed evidence regarding the impact of simultaneous use on subjective intoxication in naturalistic settings, with some studies suggesting increased subjective intoxication during co-use occasions relative to alcohol-only occasions [4] and other studies failing to find these effects [30]. While conflicting findings exist among the limited AA studies, participants consistently report increased intoxication during simultaneous use occasions, often referred to as “cross-fading” [40,41], and laboratory work under controlled conditions suggests synergistic effects of a single low dose of alcohol with a low dose of cannabis compared to an alcohol-only dose [16,42-44]. Individuals may engage in simultaneous use to enhance these experiences of positive subjective intoxication through increased alcohol consumption. Alternately, alcohol may alleviate negative affect states or reduce feelings of anxiety or physiological arousal experienced at higher levels of intoxication from delta-9 tetrahydrocannabinol (THC; the primary psychoactive cannabinoid in cannabis) [45]. Importantly, increased intoxication associated with higher rates of alcohol consumption is a robust predictor of risk for experiencing alcohol consequences [4,46-49], and may explain the link between simultaneous use and alcohol consequences, such as drinking more than intended, experiencing nausea or vomiting, or neglecting responsibilities (refer to Figure 2 and the Aim 2 section).

#### Disinhibition

There is evidence that simultaneous use, compared to either substance alone, increases disinhibition (the ability to suppress an automatic behavioral or cognitive response), a core component of executive functioning [50]. Laboratory studies suggest that both cannabis [51-53] and alcohol [54-57] acutely impair inhibitory control (one indicator of disinhibition). In line with this, research and theory suggest that one’s ability to moderate alcohol consumption is lower when inhibitory control is impaired [58,59]; this alcohol-induced impairment in inhibitory control increases ad libitum alcohol consumption [60]. Thus, simultaneous use could increase disinhibition, resulting in increases in alcohol use compared to nonsimultaneous use situations. This possibility has not been well studied in real time.

#### Craving

Cannabis and alcohol use both activate the endocannabinoid system [61] and, in turn, the approach motivation system [62], thereby increasing the tendency to approach rewarding stimuli (eg, alcohol) [63]. As alcohol and cannabis are commonly paired and known to increase the pleasurable effects of both drugs [16,64], simultaneous use may trigger an alcohol craving. Craving increases after exposure to alcohol cues and initiation [65-67], but only 1 laboratory study examined the effects of simultaneous use, finding an increase in “drug wanting” [67]. Active THC (vs placebo) alone has been shown to lower alcohol craving in a laboratory study [68], but no studies have examined the effect of simultaneous use on alcohol craving in the natural environment. AA methods are ideal for examining the direct effects of simultaneous use on the proposed mechanisms, as they can be assessed during the simultaneous use events*.*

### Social Context in Simultaneous Use

Another critical consideration for understanding how simultaneous use affects alcohol outcomes is social contexts (eg, the number of people present and the percent of people who are drinking), which are consistent predictors of a higher level of alcohol consumption [69-71]. Research on the context of alcohol consumption suggests that individual differences in motivation to drink predict drinking in certain contexts, but that social context is a consistent motivator for heavy drinking [72,73]. A recent EMA study of college students revealed that a majority of cannabis use episodes involved being with others and found a positive association between using cannabis with others and the amount of time spent using it [74], suggesting using in social contexts may lead to higher levels of cannabis consumption as well. Recent work has also examined social contexts of simultaneous use episodes, revealing that among young adults, social events in private settings with a high percentage of people who are intoxicated resulted in an increased likelihood of simultaneous use [75], and among adolescents in social contexts with a greater number of underage individuals drinking was associated with an increased likelihood of simultaneous use [46]. In daily or EMA data, social contexts were significantly associated with simultaneous use occasions (relative to alcohol or cannabis only) among college students [76] and young adult populations [77]. In qualitative work, young adults and adolescents suggest that they consider physical, social, and situational contextual factors when engaging in simultaneous use and that social characteristics are associated with simultaneous use [78,79]. Despite the converging evidence of the importance of social contexts for simultaneous use, no studies to date have examined whether context moderates the association between simultaneous use and alcohol consumption or consequences (refer to Figures 1 and 2 and the Aim 3 section).

### AA of Simultaneous Use

AA methods include passive data collection (eg, wearable biosensors) and EMAs; they are ideal for studying substance use behavior and consequences as they allow for the assessment and control of contextual and social factors integral to substance use behaviors [25], as well as timing and direct effects as they naturally occur [80,81]. AA also allows for the assessment of intoxication and impairment from self-administered doses of alcohol and cannabis and with higher concentrations of THC than cannabis available for laboratory research. Recent advancements in AA also afford the opportunity to objectively assess substance use impairment via behavioral and cognitive tasks in the natural environment [82], expanding our ability to test these mechanisms in real time as they occur.

Transdermal alcohol biosensors provide a minimally invasive, objective, and passive method for continuously assessing alcohol consumption in the natural environment via transdermal alcohol concentration (TAC), a measurement derived from the small fraction (approximately 1%) of consumed alcohol excreted through the skin [83]*.* TAC sensors show robust correlations with breath alcohol sensors in the laboratory [84-86] and provide an objective, fine-grained indicator of alcohol use and specific high-risk patterns (eg, rate of consumption) and are stable indicators of within-person variability [87,88], allowing for the examination of time-varying factors (eg, cannabis use or context) on patterns of consumption. The 3 characteristics of drinking events that can be derived from TAC data and are of particular interest to our study aims are absorption rate (a function of physiological absorption rate and behavioral factors, such as drinking pace and stomach contents), peak TAC (a proxy for maximum blood alcohol concentration or a marker of peak intoxication), and area under the curve (including time and TAC values as an approximation of volume of alcohol consumed) [84,88]. TAC data mitigates the sole reliance on subjective self-report while reducing participant burden and circumventing issues with lower compliance with AA at higher drinking rates [89]. Taken together, pairing EMA with alcohol biosensors minimizes recall bias, maximizes external validity, and enhances the ability to more precisely model the influence of proximal factors linked with alcohol outcomes in the natural environment [90].

### Study Aims and Hypotheses

This protocol aims to examine mechanisms by which simultaneous use leads to alcohol consumption and consequences using naturalistic data collection. In addition to traditional self-report EMA methods (eg, assessing self-report craving and subjective impairment), the study will leverage technological advances in AA to assess motor impairment (gait and balance) and disinhibition (inhibitory control) with app-based behavioral tasks in a participant’s natural environment, as well as alcohol biosensors to passively and objectively measure patterns of alcohol consumption. This is the first study to directly examine the mechanisms by which simultaneous use leads to increased alcohol consumption and its consequences in the natural environment. Further, it is the first study to use transdermal alcohol biosensors and behavioral measures to assess objective impairment during simultaneous use events in the natural environment. The specific aims and hypotheses presented in Textbox 1 will be examined.

**Study aims.**

**Aim 1**

Prospectively examine whether simultaneous use (vs alcohol only) is associated with increased alcohol consumption at the event level, as measured objectively and via self-report.

**Aim 1a**

Evaluate mechanisms by which simultaneous use may be associated with increased alcohol consumption. We hypothesize that simultaneous use will be associated with (1) increased disinhibition, (2) increased subjective intoxication, and (3) increased alcohol craving, and in turn increased alcohol consumption (self-report).

**Aim 1b**

Examine whether simultaneous use is associated with high-risk patterns of alcohol use (eg, increased rate of consumption), as measured objectively (transdermal alcohol concentration [TAC]).

**Aim 2**

Prospectively examine whether simultaneous use is associated with increased alcohol consequences at the daily level.

**Aim 2a**

Evaluate mechanisms that may explain the association between simultaneous use and alcohol-related consequences. We hypothesize that simultaneous use will result in increased alcohol intoxication, as measured by (1) motor impairment (gait and balance) and (2) subjective impairment, leading to a greater number of alcohol consequences.

**Aim 2b**

Examine whether simultaneous use leads to increased alcohol consequences via increased alcohol consumption, as measured objectively (TAC).

**Aim 3**

Examine event-level contextual moderators of the association between simultaneous use and alcohol consumption and consequences. We hypothesize that simultaneous use in social contexts (eg, locations where there are others drinking) relative to nonsocial contexts will result in (1) increased alcohol consumption and (2) more alcohol-related consequences.

## Methods

### Design Overview

Young adult participants (aged 18-30 years) complete a baseline session, 28 consecutive days of AA, including daily EMA and continuous wear of the BACTrack Skyn transdermal alcohol biosensor bracelet, and 2 internet-based videoconference sessions during the AA period (a midpoint check-in at 14 days and a final session at 28 days). Data collection assesses the characteristics of alcohol and cannabis use events, in addition to hypothesized mechanisms and moderators of the association between simultaneous use events and alcohol outcomes (consumption and consequences).

### Ethical Considerations

The human participants ethics review was completed and approved by the university institutional review board (IRB#1911002571). All participants completed informed consent, where all aspects of the study protocol were reviewed by a research assistant and knowledge checks were completed before a participant consented. All study data are deidentified. Monetary compensation (described in the Compensation section) was provided and deemed to be commensurate with time to complete study procedures.

### Participants

Up to 150 young adult participants will be recruited to achieve a final sample of 115 participants (accounting for up to 20% attrition) who complete the full study. Eligibility criteria include the following: (1) aged 18 to 30 years; (2) ability to read and speak English; (3) drink alcohol on average twice per week (or 16 times) and drink heavily (>5 drinks for men and >4 drinks for women per occasion) on average once per week (or 8 times) over the last 60 days; 4) and use cannabis on average of at least once weekly (or 8 times) for the past 60 days; 5) report recent (at least once in the past 30 days) simultaneous alcohol and cannabis use (defined as using both alcohol and cannabis at the same time so that their effects overlap); 6) no recent use (past 30 days) of substances other than alcohol, cannabis, or tobacco; 7) not currently in or seeking treatment for cannabis or alcohol use; 8) not experiencing suicidal ideation (ie, current intent) or symptoms of psychosis or mania in the past 30 days; 9) own a smartphone; 10) live within a 10-mile radius of study location site or willing to drive within this radius to pick up study materials; 11) not color-blind; and 12) not currently working overnight (ie, third shift).

### Procedures

#### Recruitment and Screening

**Figure 3:**
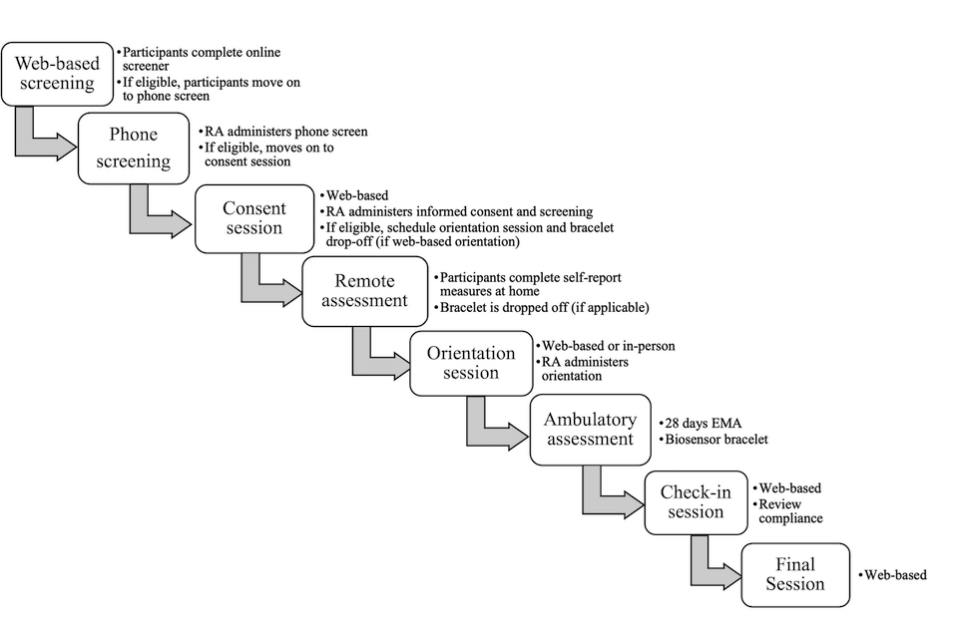
Study flow. EMA: ecological momentary assessment; RA: research assistant.

Advertisements targeting individuals who use alcohol and cannabis include print and web-based media, social media, flyers, and handouts posted in the local community, as well as “snowball” recruitment [91] (ie, participant referrals). Participants who respond to advertisements complete a web-based screener, followed by a telephone screener to assess initial eligibility. Following initial remote screening, participants are scheduled for an internet-based videoconference session in which they complete informed consent, followed by the Structured Clinical Interview for DSM-5 Research Version (SCID 5-RV) [92] interview to rule out those with current psychosis, mania, or suicidal intent and a TLFB [24] interview to confirm eligibility on past 60-day alcohol, cannabis, and other substance use. Eligible participants complete a demographic and substance use history questionnaire and are scheduled for the baseline orientation session. Figure 3 depicts the study flow.

#### Baseline Orientation Session

The baseline orientation occurs in person or via videoconference. The decision to conduct the session in person or via Zoom videoconference (Zoom Video Communications, Inc) depends on participant preference and the status of university policies around the COVID-19 pandemic. If via Zoom, research staff deliver the Skyn biosensor to the participant in advance of the orientation session. Before the session (regardless of whether it occurs in person or virtually), participants complete a web-based assessment consisting of questionnaires to assess various mental health, personality, and substance use constructs (Multimedia Appendix 1 [93-109] shows a full list of non-EMA self-report measures). During the orientation session, participants train in the use of the survey app (TigerAware; TigerAware LLC [110]), including how and when to initiate surveys, answer different survey types, estimate standard drink and cannabis quantity reporting, and when to reach out to staff during data collection. They are trained on the use of the Skyn biosensor for AA data collection, including when and how to sync and charge the bracelet and when to remove it (eg, to avoid water damage). They also receive study-related handouts detailing reminders of the study protocol, Skyn biosensor operating procedures, and personalized standard alcohol and cannabis quantity estimates.

#### The AA

##### The EMAs

Immediately following the baseline orientation session, participants begin the AA data collection phase, which consists of using their personal smartphones to answer daily surveys (EMA) and wearing the Skyn biosensor in daily life. The EMA protocol (Multimedia Appendix 1 for full EMA survey details) will instruct participants to self-initiate event-contingent surveys when they begin drinking alcohol or using cannabis (this is termed a *start survey*) and when they finish drinking or using cannabis (a *finish survey*) during each day of the AA data collection period. Participants are instructed to complete a finish survey if they are done using cannabis or drinking but forgot to initiate the start survey. Follow-up surveys at 30, 60, 90, and 120 minutes are pushed after the initiation of a start survey to capture additional use, intoxication, affect, craving, and contextual changes. Follow-up surveys assess hypothesized mechanisms (disinhibition, subjective intoxication, and craving) and social context moderators (refer to the Data Analysis section for more details).

In addition to event-contingent surveys, participants receive signal-contingent surveys (ie, random prompts) to capture substance use periods that were not self-initiated and social contexts during nonuse moments. Random prompts occur once within each 3-hour block during the afternoon, evening, and night (block 1: 3 PM-6 PM, block 2: 6 PM-9 PM, and block 3: 9 PM-12 AM). Prompts for these random surveys are not sent if they would occur during the follow-up period of a start survey. When substance use is reported on a random survey, follow-up surveys identical to those following the start surveys are prompted.

Finally, a time-contingent *morning survey* is available to be self-initiated every morning and prompted by 2 reminders (9 AM and 11 AM). Morning surveys assess the prior days’ alcohol and cannabis use, alcohol and cannabis-related consequences, other substance use, reasons for nonuse, and intentions for use in the present day. All surveys are designed to be short (<3 minutes), and participants may suspend prompts at any time to avoid interrupting their sleep or tasks (eg, employment or education activities).

##### Passive Alcohol Consumption Assessment

**Figure 4:**
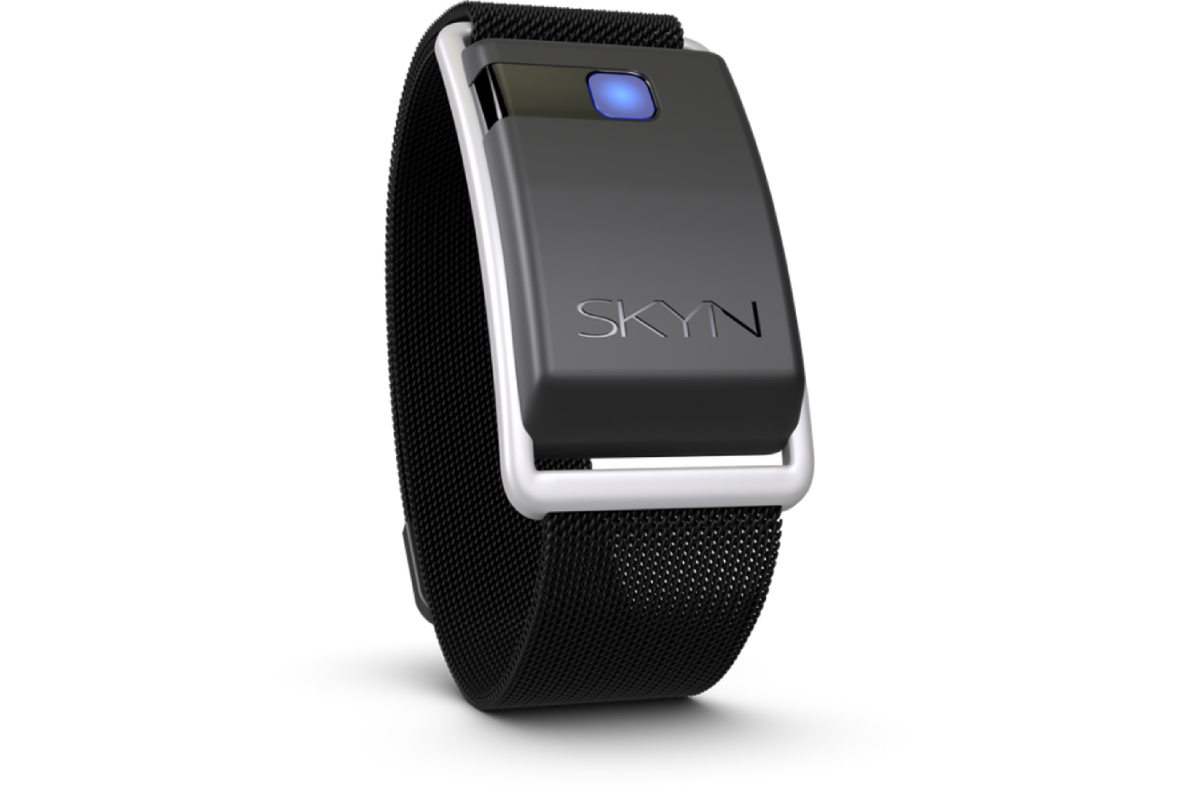
BACTrack Skyn bracelet.

The Skyn biosensor is worn continuously on the nondominant wrist of each participant to passively collect TAC during the AA data collection period (Figure 4). The app used to transmit data collected via Bluetooth from the Skyn biosensor is not supported on Android (Google LLC) devices; therefore, study iPhones (Apple Inc) are provided for syncing the Skyn biosensor only (no data or cell service required) for participants who own Android phones; study-provided phones are not used for EMA data collection. Participants are instructed to open the Skyn app and sync the app once a day, which they are compensated to complete (Tables 1 and 2). Participants are also instructed to wear the bracelet at all times but are told that it is not waterproof, so to only remove it when charging, swimming, showering, or doing any other activities where the bracelet may be submerged in water. The charging protocol varies based on the bracelet version. In September 2021, BACTrack Skyn released a new bracelet, which has a prolonged battery life (average of 13 days), relative to the previous version (48-72 hours). The protocol for charging instructions is adapted to these versions. It is emphasized during orientation to wear the bracelet while drinking and overnight, as drinking often occurs in the evening hours, and removing the bracelet before full metabolization of alcohol consumption would result in missing data. Therefore, we suggest participants that they charge the bracelet while bathing to reduce the number of removals. Participants are instructed to avoid exposure to alcohol-based products (eg, using hand sanitizer) when possible, as this can cause a temporary elevation in TAC (this does not resemble a consumed alcohol episode but is troublesome during data cleaning and should be minimized).

**Table 1:** Compliance schedule.

Reimbursement for sessions	Compensation (US $)
Orientation session	30
Remote baseline assessment	25
Check-in session	15
Final session	15
Completion bonus	50

**Table 2:** Weekly reimbursement for daily surveys^a^.

		Week 1 compensation (US $)	Week 2 compensation (US $)	Week 3 compensation (US $)	Week 4 compensation (US $)
**Reimbursement for daily surveys**					
	<25% completion	5	5	5	5
	25%-49% completion	10	10	15	15
	50%-74% completion	25	25	30	30
	75%-89% completion	35	35	45	45
	>90% completion	40	40	50	50
**Reimbursement for biosensor bracelet wearing**					
	Full compliance (US $5 per day)	0-35	0-35	0-35	0-35

^a^Total possible compliance is US $450.

##### Check-in and Final Sessions

Participants complete an internet-based check-in session via Zoom videoconference after 2 weeks of AA data collection to review compliance and allow for troubleshooting of any technical aspects of the study. Finally, participants complete a final session via Zoom after coordinating the bracelet return with research staff. In the final session, research staff review the final compensation earned and administer a brief interview to receive feedback on the protocol. The interview requests general feedback on the study protocol, comfort of wearing the bracelet, the usability of the TigerAware app, and study compensation.

#### Compensation

Participant compensation is outlined in Table 2. Compliance will be maximized in the AA study phase by compensating participants by increasing dollar amounts based on compliance rates each week. Compliance is also incentivized for wearing the Skyn biosensor bracelet with a maximum of US $35 per week (US $5 per day for keeping the bracelet charged and synced). Payments are made on reloadable debit cards. Payments for orientation completion are given directly after those sessions, while total payments are given at study completion or at the time of withdrawal (prorated).

### Measures

#### Remote Baseline Assessments

Before the orientation session, participants complete a battery of self-report measures assessing various mental health, personality, and substance use constructs using a web-based Qualtrics survey (version May 2021; Qualtrics) at home (Multimedia Appendix 1 for the full list of self-report measures). This battery is expected to take approximately 1 hour to complete.

#### Baseline Demographics and Substance Use History

This survey assesses demographic information (eg, sex at birth, gender, race, and ethnicity) and substance use history information (eg, age at first alcohol and cannabis use and history of use of substances other than alcohol and cannabis). In addition, a subset of items from the Daily Sessions, Frequency, Age of Onset, and Quantity of Cannabis Use Inventory [111] are included. The survey takes approximately 15 minutes to complete during the recruitment and screening session and was completed using a web-based Qualtrics survey by the participant. Full scoring information is not described here, as a subset of items were included in this study; however, this information is provided in the original citation for the Daily Sessions, Frequency, Age of Onset, and Quantity of Cannabis Use Inventory [111].

#### The TLFB

The TLFB [24] is a calendar-assisted interview administered by a research assistant to assess recent daily substance use. It uses a calendar to assist participants in remembering substance use, with cues for personal events and dates to enhance accurate recall. The TLFB will be used to assess past 60-day alcohol (number of drinks), cannabis, tobacco, and other substance use to confirm eligibility and characterize substance use patterns. It has been found to be reliable and valid in a variety of prior studies with various substances and populations [112-115] and takes approximately 10 to 30 minutes to complete, depending on the substance use patterns of the participant who is being interviewed.

#### The SCID 5-RV

The SCID 5-RV [92] semistructured interview administered by research staff includes a screening tool that is used to assess current symptoms of psychosis and mania. The SCID 5-RV screening tool for mania and psychosis takes approximately 10 minutes to complete and is scored based on *Diagnostic and Statistical Manual of Mental Disorders, Fifth Edition* criteria for each diagnosis. It has shown strong clinical validity and intrarater and test-retest reliability [116].

#### Patient Health Questionnaire-9

The Patient Health Questionnaire-9 [117] is a self-report measure administered by the participant of current depressive symptoms, including suicidal ideation, and is used to screen for current suicidal intent. The Patient Health Questionnaire-9 is administered using a web-based Qualtrics survey and takes approximately 5 minutes to complete. It has shown strong reliability and validity as a self-report screening tool for major depressive disorder [118].

#### EMA Survey Items

Key constructs are assessed in EMA through event-contingent (begin- and end-use alcohol and cannabis surveys [ie, start and finish], follow-ups, morning) and signal-contingent (random) surveys using TigerAware [110] software. Each construct assessed is included in Table 3, and exact items are included in Multimedia Appendix 1. Measures were selected based on hypothesized associations with the primary dependent variables of alcohol consumption and consequences. Start and finish surveys are always available and begin with an assessment of whether the participant is reporting alcohol or cannabis use (or both) and continue to additional constructs as relevant (Multimedia Appendix 1). In addition, participants are instructed to complete a “hand sanitizer/alcohol-based product” survey anytime they encounter these products, which will simply ask them to enter the time they come in contact with the product. A modified version of the Stroop [119] task and a Gait and Balance task developed by Apple ResearchKit (Apple, Inc) [120] are included in the 30- and 60-minute Follow-up surveys to assess disinhibition and motor impairment, respectively. The Stroop task asks participants to select the first letter of the name of the color that is presented (eg, b for blue), where the color of the text is displayed in the opposing color for a proportion of the trials. The Gait and Balance task instructs participants to place their phones in their pockets and walk in a straight line for 20 steps.

**Table 3:** Ecological momentary assessment constructs assessed.

Construct	Delivery	Example item
Alcohol use	ST^a^, ED^b^, FU^c^, RM^d^, and MR^e^	Have you had any alcohol since your last report?
Alcohol type	ED and MR	What type of alcoholic drinks did you have?
Alcohol start time	ST, RM, and MR	Confirm the time you started drinking.
Alcohol end time	ED and MR	What time did you finish drinking?
Alcohol quantity	ST, ED, FU, RM, and MR	How many total standard drinks did you have?
Subjective intoxication	ST, FU, ED, and RM	Rate how drunk/high you feel.
Cannabis type	ST, FU, ED, and MR	Are you using flower (i.e., plant, bud)?
Cannabis quantity	ST, FU, ED, and MR	How much flower (i.e., plant, bud) are you using?
Cannabis start time	ST, RM, and MR	Confirm the time you started using flower.
Cannabis end time	ED and MR	What time did you finish using flower yesterday?
Cannabis reasons	ST and RM	Please select the reason(s) you are using cannabis.
Cannabis mode	ED and MR	Which of the following modes did you use with flower?
Context: location	ST, RM, and FU	Where are you?
Context: activity	ST, RM, and FU	What are you doing?
Context: social	ST, RM, and FU	Are you by yourself or with others?
Context: social relationship	ST, RM, and FU	Who are you with?
Context: social use	ST, RM, and FU	Are the people (person) you are with drinking alcohol?
Nicotine use	ST and MR	Are you using any of the following nicotine products?
Craving	ST, FU, RM, ED, and MR	How strong is your urge to drink alcohol right now?
Affect	ST and ED	How much have you felt upset in the past 15 minutes?
Impulsivity	ST	I did something without really thinking it through.
Disinhibition (Stroop task)	FU^f^	Multimedia Appendix 1
Motor intoxication (gait task)	FU^g^	Multimedia Appendix 1
Alcohol or cannabis consequences	MR	Did you experience any of the following yesterday as a result of your alcohol use?
Alcohol or cannabis intentions	MR	Do you plan to drink alcohol today?
Intoxication intentions	MR	How high do you plan to get today?
Nonuse reasons	MR	Please select the reasons you did not use cannabis yesterday.
Other substance use	MR	Did you use substances other than alcohol or cannabis yesterday?

^a^ST: event-contingent begin alcohol or cannabis use or start survey.

^b^ED: event-contingent end alcohol or cannabis use or finish survey.

^c^FU: event-contingent follow-up survey.

^d^RM: signal-contingent random survey.

^e^MR: event-contingent morning report.

^f^Administered in the first follow-up only.

^g^Administerd in the second follow-up only.

#### BACTrack Skyn Alcohol Biosensor

The Skyn biosensor uses fuel cell–based sensors to continuously measure TAC, body temperature, and movement in 20-second intervals. TAC data will be used to assess the daily peak TAC and rate of consumption as reflected in the rate of absorption (refer to the Analytic Plan section). Data are synced via Bluetooth on the Skyn iOS–based app by the participant, and a single CSV file is downloaded at the completion of participation. Research staff also perform daily data tracking on the BACTrack web-based portal to check for participant compliance with the syncing and charging protocol and reach out to participants if any missing data are observed.

### Data Analysis Plan

#### Data Cleaning and Aggregation

An event-level data set will be created to examine aims 1 and 2, and a day-level data set will be created to test aim 3 hypotheses. Event rows will be derived from (1) initiation of start surveys paired with corresponding Follow-up and finish surveys, (2) random surveys and corresponding follow-up surveys (when substance use is reported), and (3) “orphan” finish surveys where participants complete a finish survey but there is no corresponding start or random survey where substance use is reported or there is another preceding finish survey. Finish surveys will be paired with start or random surveys with substance use if they are submitted 6 hours apart. A social day (6 AM-6 AM) will be used to aggregate event-level data and morning surveys for a day-level data set. Each row in the day-level data set will encompass data from the morning survey and an aggregate of relevant event-level variables.

Simultaneous use events will be defined by a self-report of alcohol and cannabis use at any point in the initial survey (start or random surveys) and associated follow-up or finish surveys. Drinking events from the Skyn biosensor will be identified and processed using the TASMAC 2.0 survey software [121]. Timestamps will be used to associate and merge TAC data with drinking events. Events with no self-reported drinking or cannabis-only use with unreported (ie, missing) drinking that are indicted from TAC data will be flagged and removed from analyses when self-reported data are necessary for analyses. Social context will be defined as events where others are around or being around others who are drinking alcohol (“Are the people (person) you are with drinking alcohol?”).

All variables will be examined descriptively and checked for assumptions of normality [122]; significantly skewed variables will be transformed or modeled appropriately. Analyses will be conducted to examine whether missing data, specifically on dependent variables, are associated with baseline characteristics (eg, demographics and alcohol use disorder). Sensitivity analyses will determine if findings are consistent in cases with significant missingness [123]. Full information maximum likelihood (FIML) estimation [124] will be used for data missing at random. FIML allows for the handling of missing data without compromising the power of large, intensive longitudinal data. After checking for missing data assumptions, FIML [125] or sequential modeling will be used in multilevel models to impute missing data using packages such as *mdmb* [126].

#### Aim 1: Examine the Impact of Simultaneous Use on Alcohol Consumption

To determine if simultaneous use leads to increased alcohol consumption, data from alcohol-only and simultaneous use events (from both event- and signal-contingent surveys) will be analyzed. In addition, 2-level linear mixed effects (LMEs) [127] will be used and will include aggregated measures of level 1 (L1) predictors (random intercepts for individuals) at level 2 to control for the effect of individual-level predictors (eg, simultaneous use), allowing for the interpretation of other L1 predictors on outcomes as purely within-person associations (eg, impact of simultaneous use on total alcohol consumption). Aim 1 models will control for subject-level covariates at level 2 (sex, age, race, and ethnicity) and time-varying covariates at L1 (time lag of cannabis use for simultaneous events, tobacco, day of the week, and other substance use). Aim 1a will use mediation to examine the effects of hypothesized mechanisms (increased disinhibition, subjective intoxication, and craving measured in follow-up surveys) between simultaneous use (vs alcohol-only use) and alcohol consumption, measured via self-report (total number of drinks consumed). Aim 1b will use LME to test the association between simultaneous (vs alcohol-only) events and drinking rate (measured via TAC absorption rate). Peak TAC will be calculated as the highest TAC recorded within an episode, area under the curve will be calculated as the sum of the area of trapezoids under the TAC curve, and absorption rate is calculated as peak TAC divided by time from the last 0 reading to peak TAC [84,87,88].

#### Aim 2: Examine the Impact of Simultaneous Use on Alcohol Consequences

To examine whether simultaneous use is associated with increased alcohol consequences, LMEs will again be used with day-level aggregate data where consequences are assessed in the morning survey and simultaneous use is defined as the incidence of simultaneous use in any event data from the prior day. The same subject- and time-varying covariates will be included as in aim 1 models. Mediation in LME will again be used to test the effects of hypothesized mechanisms: motor impairment (ie, gait or balance data) and subjective impairment (self-report) measured from follow-up surveys (2a) and total alcohol consumption measured via TAC (2b) between simultaneous use and alcohol consequences.

#### Aim 3: Examine Contextual Moderators of the Association Between Simultaneous Use and Alcohol Consumption and Consequences

To test whether social context moderates the impact of simultaneous use on increased consumption and consequences, an interaction of social context (from self-report event and signal-contingent surveys) and type of substance use episode (simultaneous vs alcohol only) will be included as predictors on total alcohol consumption and consequence models, as defined earlier.

## Results

This protocol was funded in September 2019 and received institutional review board approval in January 2020. A modified protocol to accommodate COVID-19–related disruptions in research, including a fully remote protocol, was approved in April 2021, and the first participant was enrolled in May 2021. Initial recruitment goals for the project [128] aimed to enroll 95 participants to obtain a target sample of 80, accounting for attrition. However, at the time of this submission, 118 eligible participants have been enrolled, of whom 94 (79.7%) have completed the full protocol. Of the 20 who did not complete the protocol, 12 (60%) withdrew before participating in the AA, and 8 (40%) withdrew during participation. Given the successful recruitment rate and remaining funding timeline, recruitment is ongoing at the time of this submission and is intended to continue until up to 115 total participants complete the protocol. These additional participants will allow for the probing of cross-level interactions that were underpowered based on the initial budget and recruitment projections in the funding proposal (eg, moderation of simultaneous use effects by frequency of cannabis use [129]). Data collection is expected to end by June 2024, and initial results for primary aims are expected in October 2024.

## Discussion

### Overview

This paper describes a protocol aimed at understanding the impact of simultaneous alcohol and cannabis use on alcohol consumption and consequences among nontreatment-seeking young adults in the natural environment. Given the high prevalence of simultaneous use among young adults [1-3] and its association with high-risk behaviors and alcohol-related consequences among those who use both substances [5-9], this investigation has a critical public health impact on young adults. Furthermore, although simultaneous use has been linked to alcohol-related risks, there is conflicting evidence as to the momentary impact of cannabis use on drinking [10,35]. This study is the first naturalistic observational study that will comprehensively examine the mechanisms whereby simultaneous alcohol and cannabis use may confer alcohol-related risks in the moment and across contexts. With the use of EMA and alcohol biosensors, this protocol will objectively identify how self-reported cannabis impacts patterns of drinking (eg, rate of consumption). Furthermore, the assessment of alcohol and cannabis use behaviors in the moment, while young adults are in their natural drinking environment, will allow for the direct measurement of the critical risk mechanisms under study, specifically craving, subjective intoxication, motor impairment, disinhibition, and contextual influence.

### Study Implications

The comprehensive study of these substance use patterns, and the identification of key hypothesized mechanisms are critical to informing prevention and intervention efforts. In particular, this work will inform the next wave of technology-assisted treatment and intervention research, such as just-in-time adaptive interventions [130-133] and digital interventions [134]. There has been significant interest in this approach, specifically in the use of mobile apps to reduce alcohol use [135] and several effective trials; however, no work yet has tested interventions for simultaneous use behaviors in young adults. To do this important prevention and intervention work, it is critical to understand the specific risk mechanisms of simultaneous use on alcohol outcomes. This study will elucidate these mechanisms and clarify the competing literature (ie, substitution vs complementary theories) on the momentary impact of simultaneous use on alcohol outcomes to inform these intervention approaches.

This study will also pave the way for additional work aimed at integrating alcohol biosensor assessment within intensive longitudinal research protocols using EMA as well as for preventive interventions. While this is not the first study to use wrist-worn biosensors in naturalistic data collection [136,137], this is the first to align the use of additional substances within drinking events to understand how co-use may impact drinking patterns, such as the rate of consumption. This work will not only elucidate the impact of cannabis use on drinking topography in naturalistic settings, thereby clarifying the role of simultaneous use in risky alcohol consumption patterns, but also establish the feasibility of complex polysubstance use assessment methods across levels of data analysis. This research has the potential to lead to important health policy recommendations, such as regulations on the proximity of retail cannabis and alcohol placement and the distribution of products that include both alcohol and cannabis.

### Limitations

Several limitations of the present protocol are worth identifying. First, while it is critical to understand the etiology of simultaneous use in nontreatment samples to inform prevention and developmental models of addiction, the implications of this study are limited to nontreatment-seeking young adults. Given that treatment status has been identified as a potential critical between-person moderator in the study of simultaneous use and the impact on alcohol outcomes [10], future work will be necessary to extend these findings to those with severe alcohol use disorder or who are seeking treatment for alcohol use. Second, recent use of substances other than nicotine or tobacco is an exclusion criterion in this protocol. This exclusion criterion was necessary due to the limited resources of this funding mechanism that prohibits recruiting a sample large enough to power for variability from additional substance use that may greatly impact alcohol consumption patterns. Although it will be controlled for in the current analyses, future work that includes individuals who use a variety of additional substances is needed to understand the full range of polysubstance use and its impact on alcohol consumption and its consequences. Finally, given that there is no validated objective method for assessing cannabis use in naturalistic data collection, this study is limited to the self-report of cannabis use in the context of an objective assessment of alcohol consumption. The objective measurement of both alcohol and cannabis use would further validate a rigorous approach to studying patterns of simultaneous use in the natural environment.

### Conclusions

This protocol describes the most comprehensive observational study to date of the impact of simultaneous alcohol and cannabis use on alcohol consumption and consequences in the natural environment among young adults. Data collected at the moment through self-report and passive alcohol sensing with a transdermal alcohol biosensor will elucidate competing theories as to how cannabis use while drinking impacts patterns of alcohol consumption and associated consequences. The study will also identify within- and between-person mechanisms and moderators of these key associations, such as subjective and objective measures of intoxication, via assessment during substance use events. Results will inform key prevention and intervention efforts to reduce the negative impact of simultaneous use among young adults who use alcohol and cannabis frequently and set the groundwork for future work aiming to use these methods in expanded populations.

## Appendix

Multimedia Appendix 1 This includes all measures administered at the remote assessment, as well as the full battery of ecological momentary assessment survey items.

Multimedia Appendix 2 Peer-review reports from AA-2 - Epidemiology, Prevention and Behavior Research Review Subcommittee - National Institute on Alcohol Abuse and Alcoholism Initial Review Group (National Institutes of Health, USA).

## Abbreviations

AA: ambulatory assessment
EMA: ecological momentary assessment
FIML: full information maximum likelihood
L1: level 1
LME: linear mixed effect
SCID 5-RV: Structured Clinical Interview for DSM-5 Research Version
TAC: transdermal alcohol concentration
THC: delta-9 tetrahydrocannabinol
TLFB: Timeline Followback

## Data Availability

All data, including acknowledgment of missing data, will be made available at study completion at the preregistration site [128].
