# Supplementary material for: Examining the Impact of Simultaneous Alcohol and Cannabis Use on Alcohol Consumption and Consequences: Protocol for an Observational Ambulatory Assessment Study in Young Adults
Source: JMIR Res Protoc. 2024 Sep 25;13:e58685. doi: 10.2196/58685 (PMC11464943; doi:10.2196/58685)
Supplement: Multimedia Appendix 1 [file resprot_v13i1e58685_app1.docx]

**Appendices**

**Appendix A: Full List of Remote Measures**

1. Alcohol and Cannabis Simultaneous Use Scale (ACSUS)^1^
2. Alcohol Expectancy Questionnaire- Brief^2^
3. Alcohol Purchase Task^3^
4. Alcohol Use Disorder Identification Test (AUDIT)^4^
5. Beck Anxiety Inventory^5^
6. Brief Marijuana Consequences Questionnaire (B-MACQ)^6^
7. Brief Young Adult Alcohol Consequences Questionnaire (B-YAACQ)^7^
8. Cannabis Use Disorder Identification Test (CUDIT)^8^
9. Comprehensive Marijuana Motives Questionnaire^9^
10. COVID Impact Survey^10^
11. Distress Tolerance Scale^11^
12. Drinking Motives Questionnaire^12^
13. Everyday Discrimination Scale^13^
14. Marijuana Effect Expectancy Questionnaire- Brief (MEEQ-B)^14^
15. Marijuana Purchase Task^15^
16. Self-rating of the Response to Alcohol Form (SRE)^16^
17. Simultaneous Alcohol and Marijuana Motives Questionnaire (SAMMQ)^17^
18. UPPS-short Impulsive Behavior Scale (UPPS-P)^18^

**Appendix B: Ecological Momentary Assessment Survey Details**

**Event-Contingent begin drink/cannabis (“Start”):**

1. *Which of the following are you reporting?*
   - *I started drinking*
   - *I started using cannabis*
   - *I started drinking and using cannabis*

[If drinking]:

1. *Confirm the time you started drinking.*

(time picker)

Text slide: *Reminder: this is a standard drink*

*
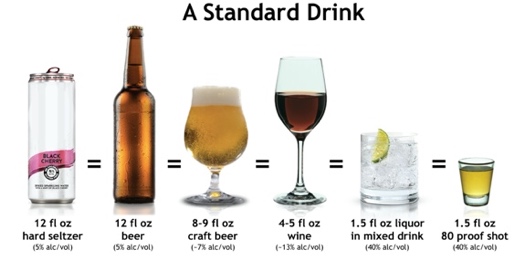
*

1. *How many total standard drinks are in your current drink?*

*Note: Include any prior drinks you forgot to report*

*1.0 drink -----------------------------10 or more drinks*

1. *Rate how drunk you feel*

*0 ______________________________________10*

*Not at all drunk As drunk as possible*

[If Cannabis]:

1. Text slide: “*For the following questions about cannabis use, report what you are currently using and any cannabis you forgot to report earlier.”*
2. *Are you using flower (i.e., plant, bud)?*

- *Yes*
- *No*

1. *Are you using concentrates (e.g., oil, wax)?*

- *Yes*
- *No*

1. *Are you using edibles?*

- *Yes*
- *No*

[IF yes to flower]:

*a) How much flower (i.e., plant, bud) are you using?*

*(****Note****: report only the cannabis you have personally consumed)*

*1/16-------------------------2.0+ grams(slider)*

Scale options: 1/16 gram, 1/8 gram, 1/4 gram, 1/2 gram, 1 gram, 1.5 grams, 2 grams, 2 + grams

[IF yes to concentrate]:

*c) How many hits of concentrate are you using?*

1-*------------------------------15 (slider)*

*Hit ----------------------------- or more hits*

[IF yes to edible]:

*e) How many mg of edible products are you using?*

*(****Note****: standard dose of edible product is 5-10mg THC)*

1mg ------------------------------------ 100 or more mg (slider)

1. *Confirm the time you started using* [repeats for each type]*.*

(time picker)

1. *Please select the reason(s) you are using cannabis.*

*(Mark all that apply)*

- - *To be social*
  - *To feel less anxious/depressed*
  - *To relieve physical pain (e.g., headache)*
  - *To sleep better*
  - *To relax*
  - *To enjoy the effects*
  - *Other reason: ________*

1. *Are you using cannabis for any of the following reasons?*

*(mark all that apply)*

- *To feel more drunk/enhance the effects of alcohol*
- *Because I was too drunk*
- *As a substitute for alcohol*
- *None of these reasons*

1. *Rate how high you feel*

*0 ____________________________________________10*

*Not at all high As high as possible*

[If report both alcohol and cannabis]:

1. *Rate how cross-faded (i.e., both drunk and high) you feel:*

*0 ____________________________________________10*

*Not at all cross-faded As cross-faded as possible*

[Context Questions]:

1. *Where are you?*

*(mark all that apply)*

- - *My home*
  - *Friend/family member’s home*
  - *Bar/club/restaurant*
  - *Outdoor*
  - *Work/school*
  - *Car*
  - *Other ___________*

1. *What are you doing?*

*(mark all that apply)*

- - *Relaxing/hanging out*
  - *Watching T.V./movie*
  - *Social media/online*
  - *Partying*
  - *Working/studying*
  - *Playing video games*
  - *Other: _____________*

1. *Are you by yourself or with others?*

- *By myself*
- *With others*

[If with others]:

- 1. *How many people are you with?*

*1________________________ 50+*

*Person or more people*

- 1. *Who are you with?*

*(mark all that apply)*

- - - *Partner/significant other*
    - *Roommate(s)*
    - *Friend(s)*
    - *Family*
    - *Stranger(s)/acquaintances*
    - *Coworker(s)/classmates(s)*
    - *Other* _____________
  1. *Are the people (or person) you are with using alcohol?*
  - *Yes, everyone (and if only one person)*
  - *Yes, most people*
  - *Yes, some people*
  - *No*
  - *I don’t know*
  1. *Are the people (or person) you are with using cannabis?*
  - *Yes, everyone (and if only one person)*
  - *Yes, most people*
  - *Yes, some people*
  - *No*
  - *I don’t know*

1. *Are you using any of the following nicotine products?*

*(mark all that apply)*

- - *Yes, cigarettes*
  - *Yes, e-cigarettes or nicotine vaporizers*
  - *Yes, other tobacco or nicotine products*
  - *None*

1. *How much have you felt [affect] in the PAST 15 MINUTES?*

*1 = very slightly or not at all*

*2 = a little*

*3 = moderately*

*4 = quite a bit*

*5 = extremely*

[Insert each of the following affect words in random order]:

*Upset*

*Nervous*

*Afraid*

*Alert*

*Determined*

*Attentive*

1. *How strong is your urge to drink alcohol right now?*

*No Urge Strongest ever*

*0_____________________________________________________10*

1. *How strong is your urge to use cannabis right now?*

*No Urge Strongest ever*

*0_____________________________________________________10*

1. *[MIS] To what extent have you felt this way in the last 15 minutes?*
   1. *I did something without really thinking it through.*

*1=very slightly or not at all*

*2=a little*

*3=moderately*

*4=quite a bit*

*5=extremely*

- 1. *I felt and acted on a strong impulse.*

*1=very slightly or not at all*

*2=a little*

*3=moderately*

*4=quite a bit*

*5=extremely*

- 1. *I gave up easily.*

*1=very slightly or not at all*

*2=a little*

*3=moderately*

*4=quite a bit*

*5=extremely*

- 1. *I did something for the thrill of it.*

*1=very slightly or not at all*

*2=a little*

*3=moderately*

*4=quite a bit*

*5=extremely*

*22) [Text slide]: Thank you for completing the survey! Remember you will receive follow-up surveys where you can report any additional use.*

**Follow-up Assessments (30,60,90,120 minutes after Start survey completion), all available for 15 minutes:**

1. *Since your last report, have you consumed any additional alcohol and/or cannabis?*

- *Yes, I have used more alcohol and cannabis*
- *I’ve used more alcohol only* [branch to alcohol questions]
- *I’ve used more cannabis only* [branch to cannabis questions]
- *I have not used any more alcohol or cannabis* [branch to context/affect questions]

[Alcohol Questions]:

Text slide: *Reminder: this is a standard drink*

*
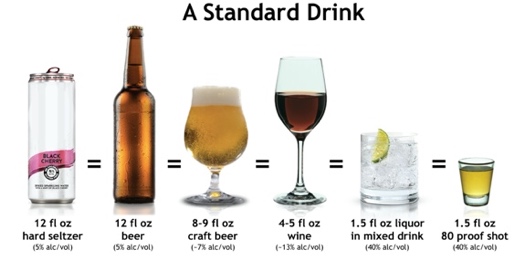
*

1. *How many additional standard drinks have you finished since your last report?*

*1.0 drink ---------------------------10 or more drinks*

1. *Rate how drunk you feel*:

*0 ____________________________________________10*

*Not at all drunk As drunk as possible*

[Cannabis Questions]:

1. *Have you used more flower (e.g., plant, bud) since your last report?*

- *Yes*
- *No*

1. *Have you used more concentrates (e.g., oil, wax) since your last report?*

- *Yes*
- *No*

1. *Have you used more edibles since your last report?*

- *Yes*
- *No*

[IF yes to flower]:

- 1. *How much flower have you used since your last report?*

*(****Note****: report only the cannabis you have personally consumed)*

*1/16 gram -------------------------2.0 or more grams(slider)*

Scale options: 1/16 grams, 1/8 gram, 1/4 gram, 1/2 gram, 1 gram, 1.5 grams, 2 grams, 2 + grams

[IF yes to concentrate]:

- 1. *How many hits of concentrate have you taken since your last report?*

1 hit-*------------------------------15 or more hits (slider)*

[IF yes to edible]:

- 1. *How many mg of edible products have you used since your last report?*

*(****Note****: standard dose of edible product is 5-10mg THC)*

1mg ------------------------------------ 100 or more mg (slider)

1. *Rate how high you feel*

*0 ____________________________________________10*

*Not at all high As high as possible*

[If report both alcohol and cannabis]:

1. Rate how cross-faded (i.e., both drunk and high) you feel:

*0 ____________________________________________10*

*Not at all cross-faded As cross-faded as possible*

[Context Questions]

1. *Have you changed locations since your last report?*
   - *Yes*
   - *No*

[If YES]:

- 1. *Where have you been since your last report?*

*(mark all that apply)*

- - - *My home*
    - *Friend/family member’s home*
    - *Bar/club/restaurant*
    - *Outside*
    - *Work/school*
    - *Car*
    - *Other ___________*
  1. *What have you been doing since your last report?*

*(mark all that apply)*

- - - *Relaxing/hanging out*
    - *Watching T.V./movie*
    - *Social media/online*
    - *Partying*
    - *Working/studying*
    - *Playing video games*
    - *Other: _____________*
  1. *Have you been by yourself or with others?*
- *By myself*
- *With others*
  - 1. *How many people have you been with?*

*0 people ________________________ 50 or more people*

- - 1. *Who have you been with?*

*(mark all that apply)*

- - - - *Partner/significant other*
      - *Roommate(s)*
      - *Friend(s)*
      - *Family*
      - *Stranger(s)/acquaintance(s)*
      - *Coworker(s)/ Classmate(s)*
      - *Other* _____________
    1. *Are the people (or person) you have been with using alcohol?*
       - *Yes, everyone (and if only one person)*
       - *Yes, most people*
       - *Yes, some people*
       - *No*
       - *I don’t know*
    2. *Are the people (or person) you have been with using cannabis?*
       - *Yes, everyone (and if only one person)*
       - *Yes, most people*
       - *Yes, some people*
       - *No*
       - *I don’t know*

1. *Have you used any nicotine products since your last report?*

*(mark all that apply)*

- - *Yes, cigarettes*
  - *Yes, e-cigarettes or nicotine vaporizers*
  - *Yes, other tobacco or nicotine products*
  - *None*

1. *How strong is your urge to drink alcohol right now?*

*No Urge Strongest ever*

*0_____________________________________________________10*

1. *How strong is your urge to use cannabis right now?*

*No Urge Strongest ever*

*0_____________________________________________________10*

1. [Stroop Task]: In the Stroop test, the participant is shown a series of words that are displayed in color and must select the first letter of the color’s name (See example screen shot below). [30-minute follow-up only]


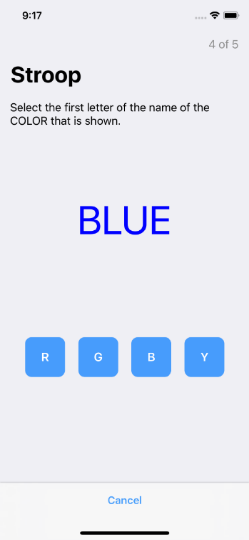


1. [5-step gait task]: In the gait and balance task, the user walks for a short distance, which may be indoors. This semi-controlled task collects objective measurements that are used to estimate stride length, smoothness, sway, and other aspects of the participant’s walking (screen shot below). [60-minute follow-up only]


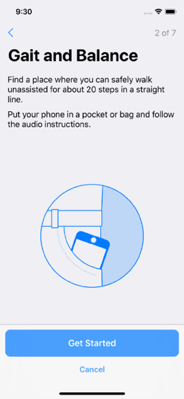


**Event-contingent finished drink/cannabis (“Finish”):**

(end of use episode, defined by participant as when they are done using alcohol or cannabis)

1. *Which of the following are you reporting?*

- *I finished drinking* [branch to finished drinking questions]
- *I finished using cannabis* [branch to finished cannabis questions]
- *I finished drinking and using cannabis* [branch to finished drinking and cannabis questions]

[If finished drinking]

1. *Confirm the time you finished drinking (i.e., when you took your last sip)?*

(time picker)

Text slide*: Reminder: this is a standard drink*

*
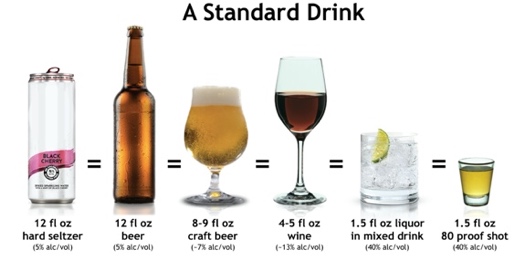
*

1. *How many total standard drinks did you have?*

*1.0 drink ---------------------------30 or more drinks*

1. *What type of alcoholic drinks did you have?*

*(mark all that apply)*

- *Beer*
- *Wine*
- *Mixed drink/cocktail*
- *Straight liquor/shot*
- *Malt beverage (e.g., hard seltzer)*
- *Other*

1. *Rate how drunk you feel*:

*0 ____________________________________________10*

*Not at all drunk As drunk as possible*

[If finished cannabis]:

1. *Confirm the time you finished using cannabis* *(i.e., took your last hit, puff, etc.)?*

(time picker)

1. *Did you use flower? (i.e., plant, bud)*

- *Yes*
- *No*

1. *Did you use concentrates (e.g., oil, wax)?*

- *Yes*
- *No*

1. *Did you use edibles?*

- *Yes*
- *No*

[IF yes to flower]:

- 1. *How much flower cannabis (in grams) did you use?*

*(****Note****: report only the cannabis you have personally consumed)*

*1/16 gram -------------------------2.0 or more grams (slider)*

Scale options: 1/16 grams, 1/8 gram, 1/4 gram, 1/2 gram, 1 gram, 1.5 grams, 2 grams, 2 + grams

- 1. *Which of the following modes did you use with flower?*

*(mark all that apply)*

- *Hand pipe/bowl*
- *Water pipe (i.e., bong)*
- *Blunt*
- *Joint*
- *Vaporizer/vape-pen*
- *Other _____*

[IF yes to concentrate]:

- 1. *How many hits of concentrate did you use?*

1 hit -*------------------------------30 or more hits (slider)*

- 1. *Which of the following modes did you use with concentrates?*

*(mark all that apply)*

- *Hand pipe/bowl*
- *Water pipe (e.g., bong)*
- *Vaporizer/vape-pen*
- *Dab-rig*
- *Other _______*

[IF yes to edible]:

- 1. *How many mg of edible products did you use?*

*(****Note****: standard dose of edible product is 5-10mg THC)*

1mg ------------------------------------ 100+ mg (slider)

1. *What is your best estimate of the THC content in the [insert product type] cannabis you used?* (repeats for each product)

- *0-10%*
- *11-20%*
- *21-30%*
- *31-40%*
- *41-50%*
- *51-60%*
- *61-70%*
- *Greater than 70%*
- *Not sure*

1. *What is your best estimate of the proportion of CBD in the cannabis you used?* (repeats for each product)
   - *Mostly/entirely CBD*
   - *Some CBD*
   - *No CBD*
   - *Unsure/I don’t know*
2. *Rate how high you feel*:

*0 ____________________________________________10*

*Not at all high As high as possible*

[Only if report finishing alcohol and cannabis use]:

1. *Rate how cross-faded (i.e., both drunk and high) you feel.*
2. *____________________________________________100*

*Not at all cross-faded As cross-faded as possible*

1. *How strong is your urge to drink alcohol right now?*

*No Urge Strongest ever*

*0_____________________________________________________10*

1. *How strong is your urge to use cannabis right now?*

*No Urge Strongest ever*

*0_____________________________________________________10*

[Affect]

1. *How much have you felt [affect] in the PAST 15 MINUTES?*
2. *= very slightly or not at all*
3. *= a little*
4. *= moderately*
5. *= quite a bit*
6. *= extremely*

[Insert each of the following affect words in random order]:

*Upset*

*Nervous*

*Afraid*

*Alert*

*Determined*

*Active*

**Signal Contingent (Random)**. Prompts will occur at random times each day within 2-hour blocks [12:00pm – 2:00pm; 3:00pm – 5:00pm; 6:00 pm – 8:00pm; and 9:00pm -11:00pm]. A single reminder prompt will be sent after 30 minutes.

*If self-initiated or follow-ups are done within the time window, random surveys will be discontinued.

**If report use on the first survey, initiate the follow-ups and cancel the rest of the scheduled in the window. ***[Time period] below will indicate “since your last report”.

1. *Have you used alcohol or cannabis since [time period]?*

- *Yes*
- *No* [Skip to Question #14]

1. *Which have you used since [time period]?*

- *I have used both alcohol and cannabis* [link to alcohol and cannabis questions]
- *I have used only alcohol* [link to alcohol questions]
- *I have used only cannabis* [link to cannabis questions]

[Alcohol questions]:

Text slide: *Reminder: this is a standard drink*

*
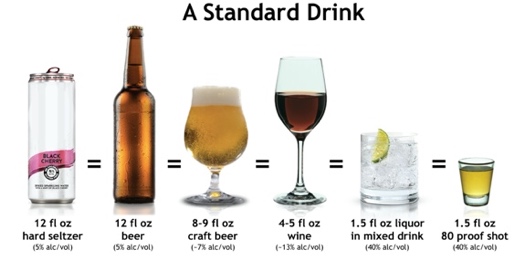
*

1. *How many standard drinks have you had since [time period]?*

***Note: Include any drinks you are currently having***

*1.0 drink --------------------------10 or more drinks (slider)*

1. *What time did you start drinking (i.e., took your first sip)?*

*(time picker)*

1. *Rate how drunk you feel:*

*0 ____________________________________________100*

*Not at all drunk As drunk as possible*

[Cannabis Questions]

1. *Have you used flower (e.g., plant, bud) since [time period]?*

- *Yes*
- *No*

1. *Have you used concentrates (i.e., oil, wax) since [time period]?*

- *Yes*
- *No*

1. *Have you used edibles since [time period]?*

- *Yes*
- *No*

[IF yes to flower]:

- 1. *How much flower cannabis have you used since [time period]?*

*(****Note****: report only the cannabis you have personally consumed)*

*1/16 gram-------------------------2.0 or more grams(slider)*

Scale options: 1/16 grams, 1/8 gram, 1/4 gram, 1/2 gram, 1 gram, 1.5 grams, 2 grams, 2 + grams

[IF yes to concentrate]:

- 1. *How many hits of concentrate have you used since [time period]?*

1 hit-*------------------------------15 or more hits (slider)*

[IF yes to edible]: *How many mg of edible products have you used since [time period]?*

*(****Note****: standard dose of edible product is 5-10mg THC)*

1mg ------------------------------------ 100 or more mg (slider)

1. *What time did you start using* *[insert product type]? (repeats for each product endorsed)*

(time picker)

1. *Which of the following describes the reason(s) you have been using cannabis since [time period]?*

*(Mark all that apply)*

- *To be social*
- *To feel less anxious/depressed*
- *To relieve physical pain (e.g., headache)*
- *To sleep better*
- *To relax*
- *To enjoy the effects*
- *Other reason: ________*

1. *Did you use cannabis for any of the following reasons since [time period]?*

*(mark all that apply)*

- *To feel more drunk/enhance the effects of alcohol*
- *Because I was too drunk*
- *As a substitute for alcohol*
- *None of these reasons*

1. *Rate how high you feel.*

*0 ____________________________________________100*

*Not at all high As high as possible*

[Only if report alcohol and cannabis use]:

1. *Rate how cross-faded (i.e., both drunk and high) you feel.*
2. *____________________________________________100*

*Not at all cross-faded As cross-faded as possible*

1. *How strong is your urge to drink alcohol right now?*

*No Urge Strongest ever*

*0_____________________________________________________10*

1. *How strong is your urge to use cannabis right now?*

*No Urge Strongest ever*

*0_____________________________________________________10*

[Context Questions, if following alcohol/cannabis reports, these questions will include phrase for “while using alcohol and/or cannabis]:

1. *Where have you been since [time period]?*

*(mark all that apply)*

- - *My home*
  - *Friend/family member’s home*
  - *Bar/club/restaurant*
  - *Outside*
  - *Work/school*
  - *Car*
  - *Other ___________*

1. *What have you been doing since [time period]?*

*(mark all that apply)*

- - *Relaxing/hanging out*
  - *Watching T.V./movie*
  - *Social media/online*
  - *Partying*
  - *Working/studying*
  - *Playing video games*
  - *Other: _____________*

1. *Are you by yourself or with others?*

- *By myself*
- *With others*

[If with others]:

- 1. *How many people are you with since [time period]?*

*1 person________________________ 50 or more people*

- 1. *Who have you been with since [time period]?*

*(mark all that apply)*

- - - *Partner/significant other*
    - *Roommate(s)*
    - *Friend(s)*
    - *Family*
    - *Stranger(s)/acquaintance(s)*
    - *Coworker(s)/classmate(s)*
    - *Other* _____________
  1. *Are the people (or person) you are/were with [time period] drinking alcohol?*
  - *Yes, everyone (and if only one person)*
  - *Yes, most people*
  - *Yes, some people*
  - *No*
  - *I don’t know*
  1. *Were the people (or person) you were with [time period] using cannabis?*
  - *Yes, everyone (and if only one person)*
  - *Yes, most people*
  - *Yes, some people*
  - *No*
  - *I don’t know*

1. *Have you used any nicotine products since [time period]?*

*(mark all that apply)*

- - *Yes, cigarettes*
  - *Yes, e-cigarettes or nicotine vaporizers*
  - *Yes, other tobacco or nicotine products*
  - *None*

1. *[Text slide only presented when alcohol or cannabis are reported]: Thank you for completing the survey! Remember you will receive follow-up surveys where you can report any additional use.*

**Morning**

1. *How many times did you remove the bracelet yesterday?*

- *Not at all*
- *Once*
- *Twice*
- *3 times or more*

[If once or more:]

- 1. *What time did you take the bracelet OFF the [first, second, third] time?*

(time picker)

- 1. *What time did you put the bracelet back ON the [first, second, third] time?*

(time picker)

- 1. *Why did you remove the bracelet the [first, second, third] time?*
     - *Bathing/Showering*
     - *Charging*
     - *Other _______*

1. *Did you use substances other than alcohol or cannabis yesterday?*
   - *Yes*
   - *No*

[IF YES]:

- 1. *Which substances did you use yesterday?*

*(Mark all that apply)*

- - - *Stimulant (e.g., Adderall, cocaine)*
    - *Opiate (e.g., oxycontin, heroin)*
    - *Sedative (e.g., benzos, Xanax)*
    - *Hallucinogen (e.g., LSD, mushrooms)*
    - *Other __________ (free response)*

1. *How strong is your urge to drink alcohol right now?*

*No Urge Strongest ever*

*0_____________________________________________________10*

1. *How strong is your urge to use cannabis right now?*

*No Urge Strongest ever*

*0_____________________________________________________10*

1. *Did you use alcohol and cannabis yesterday so that their effects overlapped?*

- *Yes, and the effects overlapped* [branch to alcohol and cannabis questions]
- *I used both alcohol and cannabis, but the effects did not overlap* [branch to alcohol and cannabis questions]
- *I only used alcohol* [branch to alcohol questions]
- *I only used cannabis* [branch to cannabis questions]
- *I did not use either alcohol or cannabis [*branch to non-use questions]

[Alcohol questions]:

Text slide: *Reminder: this is a standard drink*

*
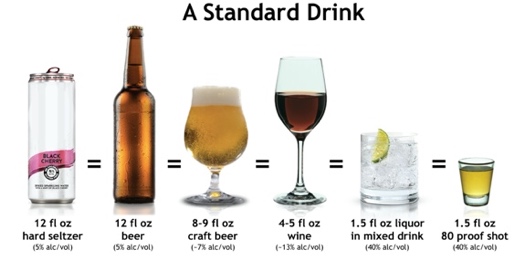
*

*14) How many total standard drinks did you have yesterday?*

*1.0 drink--------------------------------30 or more drinks (slider)*

*15) What time did you start drinking (i.e., take the first sip of your first drink) yesterday?*

*(time picker)*

*16) What time did you finish drinking (i.e., take the last sip of your last drink) yesterday?*

*(time picker)*

*17) What type of alcoholic drinks did you have yesterday?*

*(mark all that apply)*

- *Beer*
- *Wine*
- *Mixed drink/cocktail*
- *Straight liquor/shot*
- *Malt beverage (e.g. hard seltzer)*
- *Other*

[Cannabis Questions]

1. *Did you use flower (e.g., plant, bud) yesterday?*

- *Yes*
- *No*

1. *Did you use concentrates (e.g., oil, wax) yesterday?*

- *Yes*
- *No*

1. *Did you use edibles yesterday?*

- *Yes*
- *No*

[IF yes flower]:

- 1. *How much flower cannabis (in grams) did you use yesterday?*

*(****Note****: report only the cannabis you have personally consumed)*

*1/16 gram -------------------------2.0 or more grams(slider)*

Scale options: 1/16 grams, 1/8 gram, 1/4 gram, 1/2 gram, 1 gram, 1.5 grams, 2 grams, 2 + grams

- 1. *Which of the following modes did you use with flower yesterday?*
- *Hand pipe/bowl*
- *Water pipe (i.e., bong)*
- *Blunt*
- *Joint*
- *Vaporizer/vape-pen*
- *Other _____*

[IF yes concentrate]:

- 1. *How many hits of concentrate did you use yesterday?*

1 hit-*------------------------------100 or more hits (slider)*

- 1. *Which of the following modes have did you use with concentrates yesterday?*
- *Hand pipe/bowl*
- *Water pipe (e.g., bong)*
- *Vaporizer/vape-pen*
- *Dab-rig*
- *Other _______*

[IF yes edible]:

- 1. *How many mg of edible products did you use in total yesterday?*

*(****Note****: standard dose of edible product is 5-10mg THC)*

1mg ------------------------------------ 100+ or more mg (slider)

1. *What time did you start using* *[insert product type] yesterday?* [repeats for each product]

(time picker)

1. *What time did you finish using [insert product type] cannabis yesterday?* [repeats for each product]

(time picker)

[Consequences -only assessed if alcohol/cannabis use endorsed]

1. *Did you experience any of the following yesterday as a result of your* [alcohol use/cannabis use]*?*

(question repeats for alcohol and cannabis, mark all that apply)

- - *Neglected responsibilities*
  - *Got in an argument or fight*
  - *Acted rude obnoxious, or insulting*
  - *Said or did embarrassing things*
  - *Felt nauseous or vomited*
  - *Hangover*
  - *Injured self*
  - *Drove car drunk or high*
  - *Blackout*
  - *Passed out*
  - *Had difficulty concentrating*
  - *Felt lethargic or sedated*
  - *Felt depressed, sad, or anxious*
  - *Drank more alcohol than originally planned*
  - *Became more high/intoxicated than originally planned*
  - *Expressed my feelings more easily*
  - *Felt more energetic*
  - *Got a buzz*
  - *Was in a better mood*
  - *Was more sociable*
  - *Felt more relaxed*
  - *Something fun/exciting happened*
  - *Slept better*
  - *None*

1. *Did you use any nicotine products yesterday?*

*(mark all that apply)*

- - *Yes, cigarettes*
  - *Yes, e-cigarettes or nicotine vaporizers*
  - *Yes, other tobacco or nicotine products*
  - *None*

[Always received in morning report]:

1. *Do you plan to drink alcohol today?*

- *Yes*
- *No*

1. *Do you plan to use cannabis today?*

- *Yes*
- *No*

[If YES to plan to drink]

1. *How drunk do you plan to get today?*

*0 ____________________________________________100*

*Not at all drunk As drunk as possible*

[If YES to plan to use cannabis]

1. *How high do you plan to get today?*

*0 ____________________________________________100*

*Not at all high As high as possible*

[Non-use questions]:

1. *Please select the following reasons why you did not use cannabis yesterday:*[Check all that apply]

- *I was working/ had schoolwork to do yesterday*
- *I had to work/do schoolwork today*
- *I had nobody to use with*
- *I could not obtain cannabis*
- *I had no desire*
- *I wanted to feel in control*
- *I did not want to get high*
- *I was playing sports/exercising*
- *I don't usually use cannabis on this day of the week*

1. *Please select the following reasons you did not drink alcohol yesterday:*[Check all that apply]

- *I was working/ had schoolwork to do yesterday*
- *I had to work/do schoolwork today*
- *I had nobody to drink with*
- *I could not obtain alcohol*
- *I had no desire*
- *I wanted to feel in control*
- *I did not want to get drunk*
- *I was playing sports/exercising*
- *I don't usually drink on this day of the week*

**Hand Sanitizer/Alcohol-based product Report (self-initiated any time they use relevant product):**

1. *What time did you use hand sanitizer or other alcohol-based product?*

(time picker)

**References**

1. Kolp H, Horvath S, Fite PJ, et al. Development of the Alcohol and Cannabis Simultaneous Use Scale (ACSUS) in college students. *J Subst Use*. 2023;0(0):1-8. doi:10.1080/14659891.2023.2183149

2. Stein LAR, Katz B, Colby SM, et al. Validity and Reliability of the Alcohol Expectancy Questionnaire-Adolescent, Brief. *J Child Adolesc Subst Abuse*. 2007;16(2):115-127. doi:10.1300/J029v16n02_06

3. Murphy JG, MacKillop J. Relative reinforcing efficacy of alcohol among college student drinkers. *Exp Clin Psychopharmacol*. 2006;14(2):219-227. doi:10.1037/1064-1297.14.2.219

4. Saunders JB, Aasland OG, Babor TF, De JR, Fuente ’ L, Grant ’ M. Development of the Alcohol Use Disorders Identification Test (AUDIT): WHO collaborative project on early detection of persons with harmful alcohol consumption—II. *Addiction*. 1993;88:791-804.

5. Beck AT, Epstein N, Brown G, Steer RA. An Inventory for Measuring Clinical Anxiety: Psychometric Properties. *J Consult Clin Psychol*. 1988;56(893-897). doi:10.1037/0022-006X.56.6.893

6. Simons JS, Dvorak RD, Merrill JE, Read JP. Dimensions and severity of marijuana consequences: Development and validation of the Marijuana Consequences Questionnaire (MACQ). *Addict Behav*. 2012;37(5):613-621. doi:10.1016/j.addbeh.2012.01.008

7. Kahler CW, Strong DR, Read JP. Toward efficient and comprehensive measurement of the alcohol problems continuum in college students: The brief Young Adult Alcohol Consequences Questionnaire. *Alcohol Clin Exp Res*. 2005;29(7):1180-1189. doi:10.1097/01.ALC.0000171940.95813.A5

8. Adamson SJ, Kay-Lambkin FJ, Baker AL, et al. An improved brief measure of cannabis misuse: The Cannabis Use Disorders Identification Test-Revised (CUDIT-R). *Drug Alcohol Depend*. Published online 2010. doi:10.1016/j.drugalcdep.2010.02.017

9. Lee CM, Neighbors C, Hendershot CS, Grossbard JR. Development and Preliminary Validation of a Comprehensive Marijuana Motives Questionnaire. *J Stud Alcohol Drugs*. 2009;70:279-287.

10. Merikangas, K., Milham, M., Stringaris, A., Bromet, E., Colcombe, S., & Zipunnikov, V. *The Coronavirus Health Impact Survey (CRISIS). Adult Self-Report Baseline Form.*; 2020.

11. Simons JS, Gaher RM. The distress tolerance scale: Development and validation of a self-report measure. *Motiv Emot*. 2005;29(2):83-102. doi:10.1007/s11031-005-7955-3

12. Cooper ML. Motivations for Alcohol Use Among Adolescents: Development and Validation of a Four-Factor Model. *Psychol Assesment*. 1994;6(2):117-128.

13. Sternthal MJ, Slopen N, Williams DR. RACIAL DISPARITIES IN HEALTH: How Much Does Stress Really Matter?1. *Bois Rev Soc Sci Res Race*. 2011;8(1):95-113. doi:10.1017/S1742058X11000087

14. Torrealday O, Stein LAR, Barnett N, et al. Validation of the Marijuana Effect Expectancy Questionnaire-Brief. *J Child Adolesc Subst Abuse*. 2008;17(4):1-17. doi:10.1080/15470650802231861

15. Aston ER, Metrik J, MacKillop J. Further validation of a marijuana purchase task. *Drug Alcohol Depend*. 2015;152:32-38. doi:10.1016/j.drugalcdep.2015.04.025

16. Schuckit MA, Smith TL, Tipp JE. The Self-Rating of the Effects of Alcohol (SRE) form as a retrospective measure of the risk for alcoholism. *Addiction*. 1997;92(8):979-988. doi:10.1111/j.1360-0443.1997.tb02977.x

17. Patrick ME, Fairlie AM, Lee CM. Motives for simultaneous alcohol and marijuana use among young adults. *Addict Behav*. 2018;76:363-369. doi:10.1016/j.addbeh.2017.08.027

18. Cyders MA, Littlefield AK, Coffey S, Karyadi KA. Examination of a short English version of the UPPS-P Impulsive Behavior Scale ☆. *Addict Behav*. 2014;39:1372-1376. doi:10.1016/j.addbeh.2014.02.013
