# Supplementary material for: Examining the Impact of Simultaneous Alcohol and Cannabis Use on Alcohol Consumption and Consequences: Protocol for an Observational Ambulatory Assessment Study in Young Adults
Source: JMIR Res Protoc. 2024 Sep 25;13:e58685. doi: 10.2196/58685 (PMC11464943; doi:10.2196/58685)
Supplement: Multimedia Appendix 2 [file resprot_v13i1e58685_app2.pdf]

SUMMARY STATEMENT
(Privileged Communication)

PROGRAM CONTACT:
Beverly Ruffin
301-443-0281
beverly.ruffin@nih.gov

Release Date: **06/22/2019**
Revised Date:

Application Number: **1 K08 AA027551-01A1**

Principal Investigator

GUNN, RACHEL LYN

Applicant Organization: BROWN UNIVERSITY

Review Group: **AA-2**
Epidemiology, Prevention and Behavior Research Review Subcommittee

Meeting Date: **06/03/2019**
Council: **OCT 2019**
Requested Start: **09/01/2019**

RFA/PA: **PA19-117**
PCC: **AE V**
Dual PCC: **EB/HLK**
Dual IC(s): **DA**

Project Title: **Ambulatory Assessment of Simultaneous Alcohol and Marijuana Use: Impact on Alcohol Use and Consequences**
SRG Action: **Impact Score:10**
Next Steps: **Visit https://grants.nih.gov/grants/next_steps.htm**
Human Subjects: **30-Human subjects involved - Certified, no SRG concerns**
Animal Subjects: **10-No live vertebrate animals involved for competing appl.**
Gender: **1A-Both genders, scientifically acceptable**
Minority: **1A-Minorities and non-minorities, scientifically acceptable**
Age: **7A-Only Adults, scientifically acceptable**

Project Year	Direct Costs Requested	Estimated Total Cost
1	197,471	213,145
2	201,895	217,920
3	206,452	222,839
4	211,145	227,904
5	215,979	233,122
TOTAL	1,032,942	1,114,929

ADMINISTRATIVE BUDGET NOTE: The budget shown is the requested budget and has not been adjusted to reflect any recommendations made by reviewers. If an award is planned, the costs will be calculated by Institute grants management staff based on the recommendations outlined below in the COMMITTEE BUDGET RECOMMENDATIONS section.

1K08AA027551-01A1 Gunn, Rachel

SCIENTIFIC REVIEW OFFICER'S NOTES

RESUME AND SUMMARY OF DISCUSSION: The candidate of this resubmitted application requests support for training activities and research to expand her skills and knowledge on behaviors associated with co-use of alcohol and marijuana (SAM). The reviewers agreed that the applicant has resolved many of the prior weaknesses. The few minor concerns which remained (infrequent formal monitoring of progress and conceptualization of drinking context) did not impact the level of the enthusiasm. The panel unanimously agreed that the candidate is exceptional with long standing commitment (since undergraduate) to the field of addiction research. While many found the research plan ambitious, the panel members had no doubt that based on the applicant's track record Dr. Gunn "will pull it off". The reviewers unanimously believed that this proposal has a potential to produce a much-needed data on the SAM co-use and rated the submission as "Exceptional".

DESCRIPTION (provided by applicant): Through research and training activities described in this K08 proposal, the PI will acquire the skills necessary to become an independent clinical science researcher in the comprehensive study of alcohol co-use behaviors. Aside from tobacco, alcohol and marijuana are the most commonly used substances nationwide, and the most frequently co-used. With the rapid legalization of marijuana in the United States, it is likely that rates of co-use will continue to rise, particularly among young adults who currently report the highest rates of use. Despite the combined legislative changes and rising public acceptance of marijuana, little is known about how simultaneous use of alcohol and marijuana (SAM) impacts alcohol behaviors. Additionally, necessities of laboratory research (e.g. lower potency marijuana, controlled drinking contexts) present challenges in studying the effects of SAM use. The purpose of the proposed study is to examine whether SAM use leads to increased alcohol consumption and consequences by utilizing ambulatory assessment (AA) methodology. AA involves the integration of assessment methodologies (e.g. self-report, behavioral, physiological) to study individuals in their natural environment. The proposed study would be the first to employ novel behavioral assessments of disinhibition and motor impairment and biological measures of alcohol use (transdermal alcohol concentration: TAC) to study the impact of SAM use on alcohol consumption at the event level and consequences at the daily level. The study involves original data collection from young adult regular alcohol and marijuana users (N=80) who will complete 4 weeks of AA data collection while wearing alcohol biosensors to assess TAC. A combination of self-initiated and random reports of alcohol and marijuana use, subjective intoxication, contextual factors, and craving; paired with behavioral assessment of disinhibition and motor impairment (gait and balance) will be collected. The PI will collaborate with an impressive mentorship team carrying extensive experience in four areas of training: 1) behavioral and pharmacological effects of alcohol and marijuana co-use (Jane Metrik, PhD); 2) socio-contextual factors of alcohol and marijuana use (Sharon Lipperman-Kreda, PhD); 3) ambulatory assessment (Robert Miranda, PhD; Timothy Trull, PhD); 4) alcohol biosensors (Nancy Barnett, PhD); and 5) advanced statistical analyses (Kristina Jackson, PhD). The proposed 5-year training plan will prepare the PI for a career as a clinical scientist with expertise in alcohol co-use behaviors; with the goal of conducting mixed-method designs to capture behavior in controlled laboratory conditions and the natural environment. Research and training will occur at the Center for Alcohol and Addiction Studies (CAAS) at Brown University. CAAS is an interdisciplinary center devoted to research and training in the study of addiction. The proposed research will advance our understanding of mechanisms by which SAM use impacts alcohol use and consequences by building on knowledge gained from controlled laboratory research. Results will inform policy, prevention, and intervention efforts to reduce the negative outcomes associated with SAM use.

PUBLIC HEALTH RELEVANCE

Young adults (age 18-30) report the highest rates of alcohol use, alcohol use disorder, alcohol-related consequences (including mortality and morbidity), and marijuana use. The present study collects data in the natural environment to understand how simultaneous alcohol and marijuana use may impact alcohol consumption and consequences. Given the rapidly changing social and legal climate surrounding marijuana, findings from this study will inform treatment, prevention, and policy.

CRITIQUE 1

Candidate: 1
Career Development Plan/Career Goals /Plan to Provide Mentoring: 1
Research Plan: 3
Mentor(s), Co-Mentor(s), Consultant(s), Collaborator(s): 1
Environment Commitment to the Candidate: 1

Overall Impact:

Dr. Gunn is a very strong Candidate, with an already impressive publication history, strong grounding in work on the etiology of alcohol use, demonstrated interest in alcohol reaching back to her undergraduate work, and clear potential to be a successful independent researcher. The Candidate's proposed work on SAM is highly relevant to understanding the public health impact of cannabis legalization, which will be determined in large part by whether it leads to more or less alcohol use and co-use of alcohol and cannabis. The Career Development Plan includes well-chosen research activities and coursework with clear relevance to Dr. Gunn's long-term goals and a high likelihood of preparing her for independent research. The proposed research is well-conceived and likely to generate new knowledge that informs prevention and treatment of alcohol misuse. The prior research on which hypotheses and proposed procedures are based is rigorous and supports hypotheses/procedures. Some minor concerns with the Career Development Plan (e.g., formal monitoring only every 6 months) were identified. Several minor weaknesses are noted with the Research Plan (e.g., recruitment of relatively heavy drinkers, assessment of mediators at low levels of drinking). Given the overwhelming strength of the Candidate, the fact that problems were minor and addressable, the significance of the area of study, and the fact that findings are likely to inform rigorous future R01 proposals, scoring was not affected. The revised application was moderately responsive to prior critiques.

1. Candidate:

Strengths

- Dr. Gunn's productivity is impressive, with 19 publications despite having received her Ph.D. in 2017. Publications have routinely been in high quality journals, indicating a strong likelihood that future publications will be many and of high impact.
- The Candidate has demonstrated consistent interest in understanding the etiology of alcohol use. Her developing interest in SAM is a natural outgrowth of this prior work. The proposed K08 activities will build upon a strong foundation of prior experience and expertise.
- The planned training and research activities demonstrate a clear vision and understanding of the skills needed to begin a successful program of independent research.
- The mentorship team provided very strong letters of support expressing confidence that the proposed training will position Dr. Gunn for success as an independent researcher.

Weaknesses

- None noted.

2. Career Development Plan/Career Goals & Objectives:

Strengths

- The Plan includes an appropriate mix of coursework, mentored secondary data analysis, guided reading, and original research activities. These activities are well-chosen to build the desired skillset.
- A number of planned secondary analysis papers are outlined, with an achievable 2-3 papers per year planned in Years 1-4. These papers are clearly linked to the learning objectives. The topics are of importance to the field.
- The planned activities will culminate in two R01 proposals, which are clearly linked to the K08 activities and logical extensions of the learning objectives. The proposed topics of study would yield valuable information and contribute to understanding of SAM and its consequences.

Weaknesses

- 5 papers are proposed in Year 5, along with preparation/revision of two grant proposals and continuing training activities. This seems ambitious. Minor concern.
- Formal monitoring of progress now includes all members of the mentorship team, however, occurs only every 6 months. The monitoring plan remains a bit vague and plans for corrective action are not specified. This concern is very minor in light of the Candidate's prior history of success.

3. Research Plan:

Strengths

- Innovative use of TAC sensors and behavioral measures in real-world drinking contexts and elegant use of mixed methods to address gaps in prior research on SAM and alcohol craving/use.
- The study is powered to detect moderate L1 and L2 effects, as is appropriate for the funding mechanism. The plan to use repeated measures approaches maximizes the available power.
- Proposed methods and measures are well-validated and widely used and/or have been validated by the mentors in prior funded work. The Candidate will benefit from the mentors' guidance in the appropriate use, analysis, and interpretation of the included methods and measures.
- As demonstrated in section C.7, the Candidate has carefully considered alternatives to the proposed design and presents solid reasoning for the methods ultimately chosen.

Weaknesses

- Because young adults who already drink heavily (4/5+ drinks/occasion and 2+ times/week) are being recruited, there may be ceiling effects on drinking. That is, drinking may not increase much in response to SAM because it is already fairly high. Further, generalizability to adults with more normative drinking patterns may be limited.
- As in the prior submission, assessment of mechanisms ceases after consumption of 3 drinks. Reasoning for this is clear, however, concern remains that mechanisms can only be studied in the context of relatively moderate drinking. This is a minor concern for preliminary work.
- The measurement of context is clearer in the revised application; however, it remains a bit simplistic in that a few, narrowly defined aspects of context are considered. This is a minor concern for a K08 project that will build project management experience and substantive knowledge and motivate future R01 proposals.
- Issues around sex/gender as a relevant biologic variable are not well integrated into the proposal.

4. Mentor(s), Co-Mentor(s), Consultant(s), Collaborator(s):

Strengths

- The mentorship team includes highly productive and seasoned professionals with substantive expertise in areas relevant to the proposed work and to the Candidate's future plans and stated interests.
- Mentors have individually and collectively supervised a number of prior K award recipients who have gone on to receive funding.
- The addition of Dr. Lipperman-Kreda addresses prior reviewer comments about the need for a mentor with substantive expertise in drinking and SAM contexts.

Weaknesses

- None noted.

5. Environment and Institutional Commitment to the Candidate:

Strengths

- Brown University and the CAAS are excellent research environments that include all necessary supports and resources for the successful completion of the proposed training and research.
- The joint letter from the Director of CAAS and the Chair of the Department of Behavioral and Social Sciences demonstrates clear commitment to the Candidate (recommendation for a position as Assistant Professor).

Weaknesses

- None noted

Protections for Human Subjects:

Acceptable Risks and Adequate Protections

- Adequate protections are in place. The Candidate has considered risks associated with wearing a visible monitoring device and provided reasonable protections.

Data and Safety Monitoring Plan (Applicable for Clinical Trials Only):

Not Applicable (No Clinical Trials)

Inclusion Plans:

- Sex/Gender: Distribution justified scientifically
- Race/Ethnicity: Distribution justified scientifically
- For NIH-Defined Phase III trials, Plans for valid design and analysis: Not applicable
- Inclusion/Exclusion Based on Age: Distribution justified scientifically
- The exclusion of youth under age 18 is justified in that SAM use and mechanisms linking it to alcohol use are likely to differ for adolescents. The study will oversample African American/minority individuals to ensure adequate representation. Females will comprise at least 40% of the sample.

Vertebrate Animals:

- Not Applicable (No Vertebrate Animals)

Biohazards:

- Not Applicable (No Biohazards)

Resubmission:

- The authors were responsive to prior critiques. Additions/edits to the proposal have resolved many of the weaknesses identified in the prior round of review. A few minor concerns remain, including infrequent formal monitoring of progress, and a somewhat simplistic approach to the conceptualization of drinking context.

Training in the Responsible Conduct of Research:

Acceptable

Comments on Format (Required):

- The Candidate proposes an appropriate mix of online and seminar learning.

Comments on Subject Matter (Required):

- Subject matter is appropriate and extensive, and includes conflicts of interest, data management, collaborative research, research misconduct, authorship, participant recruitment, informed consent, and topics in research with vulnerable populations.

Comments on Faculty Participation (Required; not applicable for mid- and senior-career awards):

- The Candidate's main mentor and other faculty at the home institution will participate in the training.

Comments on Duration (Required):

- Appropriate

Comments on Frequency (Required):

- Appropriate. The Candidate will receive training in each year of the award period.

Select Agents:

- Not Applicable (No Select Agents)

Resource Sharing Plans:

- Unacceptable

Budget and Period of Support:

- Recommend as Requested

CRITIQUE 2

Candidate: 1
Career Development Plan/Career Goals /Plan to Provide Mentoring: 1
Research Plan: 1

Mentor(s), Co-Mentor(s), Consultant(s), Collaborator(s): 1
Environment Commitment to the Candidate: 1

Overall Impact:

This application is designed to support an exceptional candidate with a consistent record of strong productivity; clear focus and programmatic vision to establish an independent research program. The applicant's focus is the study of alcohol and marijuana co-use and its impact on alcohol-related consequences. Training objectives include the assessment of context, ambulatory assessment procedures, and. Training in the use of biosensors will contribute to the development of independence.

1. Candidate:

Strengths

- Candidate has a consistent history of productivity.
- The candidates focus on addictions goes back to her undergraduate education in which she gained experience in working with investigators on to NIAAA R01s, contributed publications, and received and NIAAA ARRA summer supplement. She has since maintained a consistent focus and trajectory through graduate education, internship, and postdoctoral study focusing on alcohol use and more recently, marijuana use.

Weaknesses

- None noted.

2. Career Development Plan/Career Goals & Objectives:

Strengths

- The training goals are closely aligned with the candidate's career objectives and will provide her with experience and expertise that will facilitate her transition to independence.
- Training goals include the development of expertise in behavioral pharmacology of alcohol and marijuana use; training in the theory and the assessment of socio-contextual factors related to marijuana and alcohol co-use; development of expertise in the ambulatory assessment of alcohol and marijuana use and co-use; and additional training in advanced statistical models.
- The career development plan includes structured meetings with mentors and a clear plan for monitoring progress.

Weaknesses

- None noted

3. Research Plan:

Strengths

- The research plan is well described and designed.
- The research plan is closely integrated with training goals.
- The research design offers a relatively comprehensive assessment of alcohol and marijuana co-use integrating biological, behavioral, and self-reported assessments.
- Feasibility of recruitment is documented based on the primary mentors existing work in this area.

Weaknesses

- None noted

4. Mentor(s), Co-Mentor(s), Consultant(s), Collaborator(s):

Strengths

- The primary mentor has extensive expertise in the study of marijuana use and has successfully incorporated protocols which provide a solid foundation for the feasibility of the proposed work.
- The mentoring team is exceptional and represents areas of expertise in all training goals.

Weaknesses

- None noted

5. Environment and Institutional Commitment to the Candidate:

Strengths

- Brown has a strong record of supporting the development of alcohol investigators. Their infrastructure is ideally suited for supporting K awards. There are clear commitments and ample resources available to support the proposed training and research.

Weaknesses

- None noted

Study Timeline:

Strengths

- Not a clinical trial

Weaknesses

- None noted

Protections for Human Subjects:

Acceptable Risks and Adequate Protections

- No concerns

Data and Safety Monitoring Plan (Applicable for Clinical Trials Only):

Not Applicable (No Clinical Trials)

Inclusion Plans:

- Sex/Gender: Distribution justified scientifically
- Race/Ethnicity: Distribution justified scientifically
- For NIH-Defined Phase III trials, Plans for valid design and analysis: Not applicable
- Inclusion/Exclusion Based on Age: Distribution not justified scientifically
- No concerns

Training in the Responsible Conduct of Research:

Acceptable

Comments on Format (Required):

- Multiple seminars and CITI

Comments on Subject Matter (Required):

- detailed and relevant

Comments on Faculty Participation (Required; not applicable for mid- and senior-career awards):

- Planned discussion with Dr. Metric and Miranda

Comments on Duration (Required):

- Continuous

Comments on Frequency (Required):

- Ongoing

Select Agents:

- Not Applicable (No Select Agents)

Resource Sharing Plans:

Unacceptable

- No data sharing plan.

Budget and Period of Support:

- Recommend as Requested

CRITIQUE 3

Candidate: 1
Career Development Plan/Career Goals /Plan to Provide Mentoring: 1
Research Plan: 2
Mentor(s), Co-Mentor(s), Consultant(s), Collaborator(s): 1
Environment Commitment to the Candidate: 1

Overall Impact:

This is an amended Mentored Clinical Science Award application (K08) from a very promising early-stage clinical researcher who seeks training and research experiences to establish an independent program of research investigating the nature and consequences of simultaneous use of alcohol and marijuana (SAM). The strengths of the application include: 1) the candidate, who has an excellent record of publication and a longstanding commitment to the field; 2) a well thought out and ambitious training plan; 3) the timely significance of investigating SAM; 4) an ambitious research plan that is well integrated with the training plan, takes advantage of mentor expertise, and has the potential to produce important data on the nature of SAM; and 5) an outstanding mentorship team. The candidate is also to be commended in her comprehensive and effective response to issues raised in the previous review. Any remaining concerns are being very minor.

1. Candidate:

Strengths

- The candidate received her Ph.D. in Clinical Science in 2017 from Indiana University, after which she began a NIAAA-sponsored postdoctoral fellowship at the Center for Alcohol and Addiction Studies (CAAS) at Brown University. She has shown a long-term commitment to a career in alcohol and addictions research that dates from her undergraduate days at the University of Kentucky.
- The candidate has considerable experience in alcohol-related studies, which has allowed her to develop expertise in a range of areas including laboratory studies and advanced statistical techniques as well as substantive knowledge about the role of impulsive behaviors and working memory in substance use behavior.
- The candidate has an excellent publication record. My Bibliography page lists 19 publications, on 9 of which she is the first author. She has consistently published in good outlets and, most significantly, all of her publications concern substance use, abuse or related behavioral disorders.
- The K08 application includes an especially strong set of supporting letters that all characterize the candidate as a 'rising star' in the field,

Weaknesses

- None noted.

2. Career Development Plan/Career Goals & Objectives:

Strengths

- The training plan is organized around a clearly articulated long-term career goal to establish an independent research career investigating the origins and consequences of simultaneous use of multiple drugs of abuse. The five training sub-goals are all relevant and clearly described and, if achieved, should provide the candidate with a solid foundation for achieving her career goal.
- For each of the five-individual training, both mentored and didactic activities are proposed goals. Although this is an ambitious training proposal that will extend throughout all five years of the K award, the individual activities are clearly described and well-integrated with the candidate's career goals as well as with the expertise of the mentorship team.
- The training proposal establishes a schedule and goals for scientific publication and grant-writing.
- In response to concerns raised in the previous review, the candidate added a fifth training goal on socio-contextual factors and also now proposes a bi-annual meeting with the mentorship team to evaluate training progress formally.

Weaknesses

- None noted.

3. Research Plan:

Strengths

- There is a pressing need to understand better the impact marijuana use has on drinking behavior and its consequences given the increasing number of US states and other political entities legalizing recreational marijuana use along with the fact that individuals who use marijuana often simultaneously use alcohol. The proposed research aimed at determining

whether marijuana use leads to higher levels of alcohol use, and if so how, is highly significant and should have important clinical implications.

- The application provides a convincing review of the existing research literature to motivate the proposed research.
- An extremely ambitious assessment protocol will produce a rich and complex dataset. Although the demands of collecting and analyzing such a dataset might typically be cause for concern, especially given the junior-level status of the principal investigator, the application provides a careful justification of the protocol, including a discussion of possible alternatives that were considered, and appropriate methods of analysis of proposed. Moreover, the candidate will be assisted by an outstanding mentorship team, with considerable experience and expertise undertaking research like that which is proposed.
- Although the protocol is demanding for participants, requiring a 5-week commitment, the applicant addressed concerns over feasibility raised in the previous review by noting that co-mentor Metrik has successfully recruited a similar sample in a more demanding protocol and that she will use similar procedures in her study to minimize attrition.
- In response to concerns raised in the previous review, the candidate has: 1) provided additional discussion of the importance of contextual factors and incorporated assessment of some contextual factors into the protocol; 2) clarified issues surrounding how alcohol and marijuana consumption will be assessed; 3) provided details on the basic psychometric properties of the scales to be used; and 4) addressed some concerns involving the proposed methods of analyses (e.g., link function to be used, consideration of gender.)

Weaknesses

- Other than some continued concern over the feasibility of undertaking such a challenging protocol, there are no additional concerns.

4. Mentor(s), Co-Mentor(s), Consultant(s), Collaborator(s):

Strengths

- The candidate has identified an outstanding set of five mentors with complementary expertise covering the five specified training domains. These include: 1) Dr. Jane Metrick (the primary mentor), an expert in the consequences and pharmacology of marijuana; 2) Dr. Robert Miranda, an expert in EMA methods for drinking and marijuana use; 3) Dr. Kristina Jackson, an expert in the analysis of complex longitudinal datasets and with the socio-contextual factors associated with substance use; 4) Dr. Nancy Barnett, an expert in the SCRAM technology to be used; and 5) Dr. Timothy Trull, an expert in ambulatory assessment methods including daily dairies and EMA.
- All five mentors are recognized as leading researchers in their respective fields, have a record of successfully mentoring the careers of both pre-doctoral and post-doctoral trainees, and have strong records of external funding through NIAAA.
- The applicant addressed concerns raised in the previous review about whether she would have adequate access to each of the five proposed mentors.

Weaknesses

- One minor concern is that the proposed mentorship team is large, consisting of five members, which may make it difficult to achieve mentor consensus. On research and training directions.

5. Environment and Institutional Commitment to the Candidate:

Strengths

- The Center for Alcohol and Addiction Studies (CAAS) at Brown University is one of the pre-eminent researches and training institutions in the addictions field. It is an ideal location to undertake the proposed research and to foster the candidate's career development.
- CAAS provides a strong research infrastructure along with a rich intellectual environment for a young addiction's researcher.
- The application includes a strong letter of support from Dr. Peter Monti, Director of CAAS. This letter conveys the institution's strong commitment to the candidate's career development.

Weaknesses

- None noted.

Protections for Human Subjects:

- Acceptable Risks and Adequate Protections

Inclusion of Women, Minorities and Children:

- Sex/Gender: Distribution justified scientifically
- Race/Ethnicity: Distribution justified scientifically
- For NIH-Defined Phase III trials, Plans for valid design and analysis: Not applicable
- Inclusion/Exclusion of Children under 18: Excluding ages <18; justified scientifically

Training in the Responsible Conduct of Research:

Acceptable

Comments on Format (Required):

- A range of formats will be used including seminars, on-line training modules, ethics courses and one-on-one meetings with mentors.

Comments on Subject Matter (Required):

- A range of appropriate topics with a focus on substance abuse related items.

Comments on Faculty Participation (Required; not applicable for mid- and senior-career awards):

- Faculty will meet with training, one-on-one, as well as offer seminars.

Comments on Duration (Required):

- Throughout the entire 5 years of the award

Comments on Frequency (Required):

- Very comprehensive

Resource Sharing Plans:

Unacceptable

- No plan provided

Budget and Period of Support:

- Recommended budget modifications or possible overlap identified:

Additional Comments to Applicant (Optional)

THE FOLLOWING SECTIONS WERE PREPARED BY THE SCIENTIFIC REVIEW OFFICER TO SUMMARIZE THE OUTCOME OF DISCUSSIONS OF THE REVIEW COMMITTEE, OR REVIEWERS' WRITTEN CRITIQUES, ON THE FOLLOWING ISSUES:

PROTECTION OF HUMAN SUBJECTS: ACCEPTABLE

INCLUSION OF WOMEN PLAN: ACCEPTABLE

INCLUSION OF MINORITIES PLAN: ACCEPTABLE

INCLUSION OF CHILDREN PLAN: ACCEPTABLE

COMMITTEE BUDGET RECOMMENDATIONS: The budget was recommended as requested.

SCIENTIFIC REVIEW OFFICER'S NOTES:

- The application does not include in detail justification for inclusion/exclusion of human subjects across lifespan as specified in NOT-OD-18-006.
- The application does not include details on plans to submit human subjects related data to NIAAA data repository as outlined in NOT-AA-18-010.
- The application does not include resources sharing plan.

Footnotes for 1 K08 AA027551-01A1; PI Name: Gunn, Rachel Lyn

NIH has modified its policy regarding the receipt of resubmissions (amended applications). See Guide Notice NOT-OD-14-074 at http://grants.nih.gov/grants/guide/notice-files/NOT-OD-14-074.html. The impact/priority score is calculated after discussion of an application by averaging the overall scores (1-9) given by all voting reviewers on the committee and multiplying by 10. The criterion scores are submitted prior to the meeting by the individual reviewers assigned to an application, and are not discussed specifically at the review meeting or calculated into the overall impact score. Some applications also receive a percentile ranking. For details on the review process, see http://grants.nih.gov/grants/peer_review_process.htm#scoring.

MEETING ROSTER

Epidemiology, Prevention and Behavior Research Review Subcommittee
National Institute on Alcohol Abuse and Alcoholism Initial Review Group
NATIONAL INSTITUTE ON ALCOHOL ABUSE AND ALCOHOLISM
AA-2
06/03/2019

Notice of NIH Policy to All Applicants: Meeting rosters are provided for information purposes only. Applicant investigators and institutional officials must not communicate directly with study section members about an application before or after the review. Failure to observe this policy will create a serious breach of integrity in the peer review process, and may lead to actions outlined in NOT-OD-14-073 at https://grants.nih.gov/grants/guide/notice-files/NOT-OD-14-073.html and NOT-OD-15-106 at https://grants.nih.gov/grants/guide/notice-files/NOT-OD-15-106.html, including removal of the application from immediate review.

CHAIRPERSON(S)

ZEMORE, SARAH, PHD
SENIOR SCIENTIST
CENTER ASSOCIATE DIRECTOR
DIRECTOR OF TRAINING
ALCOHOL RESEARCH GROUP
EMERYVILLE, CA 94608

MEMBERS

BAILEY, JENNIFER A, PHD *
RESEARCH SCIENTIST
SCHOOL OF SOCIAL WORK
UNIVERSITY OF WASHINGTON
SEATTLE, WA 98115

CAETANO, RAUL, PHD, MD, MPH *
SENIOR RESEARCH SCIENTIST
PREVENTION RESEARCH CENTER
PACIFIC INSTITUTE FOR RESEARCH AND EVALUATION
OAKLAND, CA 94612

DILILLO, DAVID, PHD *
PROFESSOR
DEPARTMENT OF PSYCHOLOGY
UNIVERSITY OF NEBRASKA-LINCOLN
LINCOLN, NE 68588

FURR-HOLDEN, C. DEBRA, PHD
C.S. MOTT ENDOWED PROFESSOR OF PUBLIC HEALTH
PROFESSOR
DEPARTMENT OF EPIDEMIOLOGY AND BIOSTATISTICS
COLLEGE OF HUMAN MEDICINE
MICHIGAN STATE UNIVERSITY
FLINT, MI 48502

GIZER, IAN ROBERT, PHD
ASSOCIATE PROFESSOR
DEPARTMENT OF PSYCHOLOGICAL SCIENCES
UNIVERSITY OF MISSOURI, COLUMBIA
COLUMBIA, MO 65211

JOHNSON, DAWN M, PHD *
ASSISTANT PROFESSOR
DEPARTMENT OF PSYCHOLOGY
UNIVERSITY OF AKRON
AKRON, OH 44325

KOSS, MARY PEASE, PHD *
PROFESSOR
DIVISION OF HEALTH PROMOTION SCIENCES
UNIVERSITY OF ARIZONA
TUCSON, AZ 85719

MCGUE, MATTHEW K., PHD
PROFESSOR
DEPARTMENT OF PSYCHOLOGY
MEMBER, INSTITUTE OF HUMAN GENETICS
UNIVERSITY OF MINNESOTA
MINNEAPOLIS, MN 55455

NEIGHBORS, CLAYTON, PHD
PROFESSOR AND DIRECTOR
DEPARTMENT OF PSYCHOLOGY
UNIVERSITY OF HOUSTON
HOUSTON, TX 77204

ONDERSMA, STEVEN J, PHD *
PROFESSOR
MERRILL PALMER SKILLMAN INSTITUTE
SCHOOL OF MEDICINE
WAYNE STATE UNIVERSITY
DETROIT, MI 48236

PATRICK, MEGAN ELIZABETH, PHD
RESEARCH PROFESSOR
INSTITUTE FOR TRANSLATIONAL RESEARCH
& INSTITUTE OF CHILD DEVELOPMENT
UNIVERSITY OF MINNESOTA
MINNEAPOLIS, MN 55415

READ, JENNIFER P., PHD
PROFESSOR
DEPARTMENT OF PSYCHOLOGY
UNIVERSITY AT BUFFALO
THE STATE UNIVERSITY OF NEW YORK
BUFFALO, NY 14260

SANCHEZ, MARIANA, PHD *
ASSISTANT PROFESSOR
DEPARTMENT OF HEALTH PROMOTION AND DISEASE
PREVENTION
FLORIDA INTERNATIONAL UNIVERSITY
MIAMI, FL 33199

SUBICA, ANDREW MAKOTO, PHD *
ASSISTANT PROFESSOR
SOCIAL MEDICINE, POPULATION, AND PUBLIC HEALTH
UNIVERSITY OF CALIFORNIA
RIVERSIDE, CA 92521

SCIENTIFIC REVIEW OFFICER

GHAMBARYAN, ANNA, MD, PHD
SCIENTIFIC REVIEW OFFICER
EXTRAMURAL PROJECT REVIEW BRANCH
OFFICE OF EXTRAMURAL ACTIVITIES
NATIONAL INSTITUTE ON ALCOHOL ABUSE AND ALCOHOLISM
NATIONAL INSTITUTES OF HEALTH
BETHESDA, MD 20892

* Temporary Member. For grant applications, temporary members may participate in the entire meeting or may review only selected applications as needed.

Consultants are required to absent themselves from the room during the review of any application if their presence would constitute or appear to constitute a conflict of interest.
